# Affinity Proteomics Reveals Elevated Muscle Proteins in Plasma of Children with Cerebral Malaria

**DOI:** 10.1371/journal.ppat.1004038

**Published:** 2014-04-17

**Authors:** Julie Bachmann, Florence Burté, Setia Pramana, Ianina Conte, Biobele J. Brown, Adebola E. Orimadegun, Wasiu A. Ajetunmobi, Nathaniel K. Afolabi, Francis Akinkunmi, Samuel Omokhodion, Felix O. Akinbami, Wuraola A. Shokunbi, Caroline Kampf, Yudi Pawitan, Mathias Uhlén, Olugbemiro Sodeinde, Jochen M. Schwenk, Mats Wahlgren, Delmiro Fernandez-Reyes, Peter Nilsson

**Affiliations:** 1 SciLifeLab Stockholm, School of Biotechnology, KTH-Royal Institute of Technology, Stockholm, Sweden; 2 Division of Parasitology, Medical Research Council National Institute for Medical Research, London, United Kingdom; 3 Department of Medical Epidemiology and Biostatistics, Karolinska Institutet, Stockholm, Sweden; 4 Department of Paediatrics, College of Medicine, University of Ibadan, University College Hospital, Ibadan, Nigeria; 5 Department of Haematology, College of Medicine, University of Ibadan, University College Hospital, Ibadan, Nigeria; 6 Childhood Malaria Research Group, University College Hospital, Ibadan, Nigeria; 7 Department of Immunology, Genetics and Pathology, Rudbeck Laboratory, Uppsala University, Uppsala, Sweden; 8 Department of Microbiology, Tumour and Cell Biology, Karolinska Institutet, Stockholm, Sweden; 9 Brighton & Sussex Medical School, Sussex University, Brighton, United Kingdom; Albert Einstein College of Medicine, United States of America

## Abstract

Systemic inflammation and sequestration of parasitized erythrocytes are central processes in the pathophysiology of severe *Plasmodium falciparum* childhood malaria. However, it is still not understood why some children are more at risks to develop malaria complications than others. To identify human proteins in plasma related to childhood malaria syndromes, multiplex antibody suspension bead arrays were employed. Out of the 1,015 proteins analyzed in plasma from more than 700 children, 41 differed between malaria infected children and community controls, whereas 13 discriminated uncomplicated malaria from severe malaria syndromes. Markers of oxidative stress were found related to severe malaria anemia while markers of endothelial activation, platelet adhesion and muscular damage were identified in relation to children with cerebral malaria. These findings suggest the presence of generalized vascular inflammation, vascular wall modulations, activation of endothelium and unbalanced glucose metabolism in severe malaria. The increased levels of specific muscle proteins in plasma implicate potential muscle damage and microvasculature lesions during the course of cerebral malaria.

## Author Summary

Why do some malaria-infected children develop severe and lethal forms of the disease, while others only have mild forms? In order to try to find potential answers or clues to this question, we have here analyzed more than 1,000 different human proteins in the blood of more than 500 malaria-infected children from Ibadan in Nigeria, a holoendemic malaria region. We identified several proteins that were present at higher levels in the blood from the children that developed severe malaria in comparison to those that did not. Some of the most interesting identified proteins were muscle specific proteins, which indicate that damaged muscles could be a discriminatory pathologic event in cerebral malaria compared to other malaria cases. These findings will hopefully lead to an increased understanding of the disease and may contribute to the development of clinical algorithms that could predict which children are more at risks to severe malaria. This in turn will be of high value in the management of these children in already overloaded tertiary-care health facilities in urban large densely-populated sub-Saharan cities with holoendemic malaria such as in the case of Ibadan and Lagos.

## Introduction

Human malaria is a life-threatening disease causing an estimated 655,000 deaths in 2010 [1]. Although the mortality rates have decreased during the last decade, deaths in Africa due to childhood malaria are still elevated with *Plasmodium falciparum* attributable to a third of the childhood deaths accounted in Nigeria [2]. Complications may develop abruptly and may be fatal. Although the most common severe syndromes, i.e. cerebral malaria, severe malaria anemia or respiratory distress, have been widely investigated, many aspects of their pathogenesis remain elusive. Furthermore, it is yet unknown what predetermines which children are at risk of developing complications.

Parasitized red blood cells (pRBC) are specifically withdrawn from the peripheral circulation during severe malaria infection through binding to and activation of vascular endothelial cells, erythrocytes, leukocytes and platelets, which may obstruct the blood flow. It is known that increased micro-vascular congestion accompanies coma in cerebral malaria and the depth of coma is correlated to the extent of the sequestration of the pRBC [3]. Plasma proteins are involved in the adhesive events of pRBC [4]–[6] and an electron-dense fibrillar material composed of immunoglobulins, fibrinogen and albumin was found deposited on vessels at autopsy and also involved in mediating adhesion of pRBC [6], [7]. In the case of severe malaria anemia, increased destruction of pRBC and non-pRBC, splenic sequestration of RBC and dyserythropoiesis contribute to anemia and free-heme-induced oxidative stress [8]. In addition, there is compelling evidence that prolonged pro-inflammatory response and an inadequate anti-inflammatory response might contribute to persistent anemia [8]. However, due to different observations in different cohort studies in cerebral malaria [9], [10], the role of circulatory inflammatory cytokines in malaria physiopathology remains elusive.

Despite the current plethora of new technologies available for analyzing and profiling proteins in body fluids, the yield of validated biomarker molecules remains low [11]. Previous studies have tried to detect protein signatures specific to malaria disease but the wide dynamic range of plasma proteins has been a limiting factor [12]–[14]. The technical issues have not yet allowed for comprehensive studies of circulating proteins since this proteome has many members and their identification is laborious. Here, we have overcome some of these challenges by applying a single-antibody microsphere-based multiplex assay utilizing more than 1,000 antibodies from the Human Protein Atlas (HPA) project [15]. For the generation of antibody suspension bead arrays, HPA antibodies are coupled to color-coded magnetic microspheres and combined to create a 384-plex-bead array. After combination with biotinylated samples, bead identity and captured plasma proteins are detected using a flow cytometric analyzer. Previously, we have shown that limits of detection reach into lower ng/ml or higher pg/ml ranges while consuming less than 1 µl of plasma sample [16] for the profiling of 384 proteins [17]. The potential to screen hundreds of analytes in hundreds of patient samples simultaneously in one parallel assay allows for an effective exploration of potential candidates in a time-efficient manner.

Here, we have employed an affinity proteomics approach to study 1,015 human proteins with 1,132 antibodies in the peripheral blood of children from Nigeria with different syndromes from severe to uncomplicated malaria as well as non-diseased parasite-negative children from the same community. Protein markers of inflammation, endothelial activation, platelet adhesion, vaso-modulation, glucose metabolism, oxidative stress and muscle-damage were found in relation to severe malaria. In particular, three muscle derived proteins, creatine kinase, carbonic anhydrase III and myoglobin, were detected in the plasma of children with cerebral malaria suggesting deep lesions into their micro-vasculature including the vascular smooth-muscle cell-layer and extra-vascular striated muscles cells, concurrent with excessive sequestration of pRBC throughout the human body. As a consequence we have identified protein signatures that allowed the distinction between the different presentations of the disease with AUCs up to 0.90 in plasma samples from a verification cohort. The data contributes to a deeper understanding of the complex mechanisms that lead to severe disease and may serve as a basis for the development of novel diagnostic strategies that would enable the prediction of the severity of malaria.

## Results

**Figure 1:**
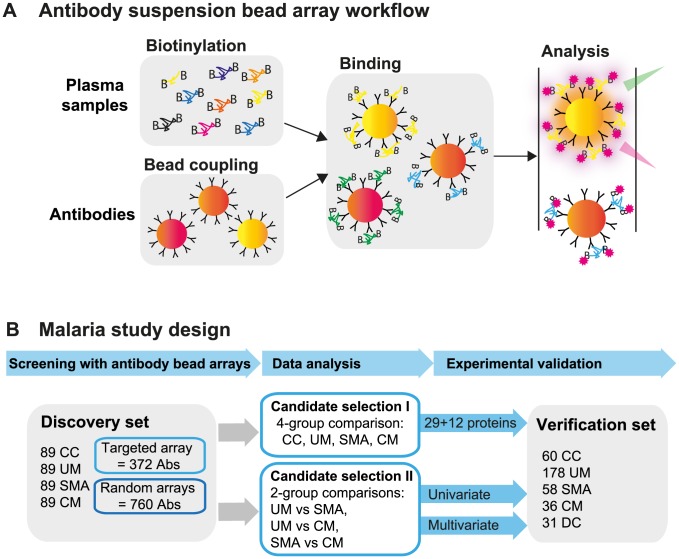
Overview of affinity proteomics screening and study design. (**A**) Schematic overview of the affinity proteomics approach using antibody suspension bead arrays. Plasma samples were biotinylated, antibodies were coupled to color-coded magnetic beads, and both were combined for analysis. Bead identity and captured plasma proteins were then detected using a flow cytometric analyzer. (**B**) Experimental design of study. Initial screening with 1,132 antibodies from targeted and blinded selections was performed in the discovery cohort (n = 356). Data from the patient groups were compared using univariate tests and multivariate penalized regression models. Identified single proteins and multi-component protein panels discriminating the 3 disease groups were validated in the verification cohort (n = 363).

In order to explore the potential of the human proteins in plasma to predict disease status during malaria infections, we have conducted an extensive exploratory profiling approach using antibody suspension bead arrays (Fig. 1A). Using two different approaches for target protein selection, with 304 unique proteins in a targeted array with carefully selected proteins and 711 in a random approach, a total of 1,015 human proteins have been profiled in 719 blood samples from different patients aiming to identify and verify protein signatures in plasma associated to the severity of malaria infection (Fig. 1B).

### Study design and selection of participants

First, a set of patients was selected and carefully matched and balanced to contain 356 childhood malaria patients and controls recruited during 2006 to 2011 in Ibadan, Nigeria. This set, denoted as “discovery cohort” in this study, included samples from patients suffering from uncomplicated malaria (UM, n = 89), severe malaria anemia (SMA, n = 89) and cerebral malaria (CM, n = 89) and from parasite-negative community controls (CC, n = 89; Table 1a). To confirm the validity of the protein signatures identified, a second set of plasma samples was analyzed including 332 independent individuals recruited from the 2009 to 2012 period. This set consisted of 178 UM, 58 SMA, 36 CM, 60 CC and 31 disease controls (DC) and was here denoted as “verification cohort” (Table 1b). The discovery and verification cohorts had 24 patient samples in common to assess the technical quality of the data in independent analyses (Methods S1 and Fig. S1C in Text S1).

**Table 1:** Clinical patient characteristics.

Table 1a. Discovery cohort patient characteristics					
Variable	Characteristics	CC (n = 89)	UM (n = 89)	SMA (n = 89)	CM (n = 89)
Sex (n, %)	female	48 (53%)	30 (33%)	36 (40%)	45 (50%)
	male	41 (46%)	59 (66%)	53 (59%)	44 (49%)
Age (month)	Mean ± SD	61±42	47±36	36±27	56±35
PCV (%)	Median	35	30	13	25
MP Density (MP/µL)	Mean	0	42.7*10^3^	38.5*10^3^	41.4*10^3^
	Unknown	0 (0%)	0 (0%)	n = 2 (2%)	n = 7 (8%)

Table 1b. Verification cohort patient characteristics						
Variable	Characteristics	CC (n = 60)	UM (n = 178)	SMA (n = 58)	CM (n = 36)	DC (n = 31)
**Sex (n, %)**	**female**	32 (53%)	68 (38%)	23 (39%)	15 (41%)	17 (53%)
	**male**	28 (46%)	108 (60%)	35 (60%)	21 (58%)	14 (46%)
**Age (month)**	**Mean ± SD**	59±41	58±38	34±24	58±34	51±35
**PCV (%)**	**Median**	34	31	14	27	29
**MP Density (MP/µL)**	**Mean**	0	49554	59629	64857	0
	**Unkown**	0 (0%)	2 (1%)	0 (0%)	0 (0%)	0 (0%)

MP: Malaria parasite.

PCV: Packed Cell Volume.

CC: Community controls, UM: Uncomplicated malaria.

SMA: Severe malaria anemia; CM: Cerebral malaria.

### Antibody selection and array design

Affinity proteomic arrays require that the analytes of interest are defined prior to analysis. For the presented approach, an inclusive and generous target selection strategy was employed by thorough literature mining of major processes previously associated to various aspects of malaria. Antibodies towards these targets were obtained based upon availability within the Human Protein Atlas (HPA). In total, a list of 372 antibodies targeting 304 different human proteins was compiled and denoted as ‘targeted array’. This list comprised primarily plasma proteins associated with acute inflammation (Fig. S9 in Text S1), iron metabolism, oxidative stress, endothelial activation, coagulation, complement activation, angiogenesis, hematopoiesis and brain injury. Two additional sets of 380 antibodies were used to profile all samples in the discovery cohort and are denoted as ‘random arrays’.

The two additional sets of antibodies were randomly chosen from the routine antibody production within the Human Protein Atlas (HPA) where more than 40,000 antigen purified and protein microarray validated antibodies have been generated. The antibodies fulfill the quality criteria of having a concentration that is higher than 50 µg/ml and that the specificity is validated on planar protein arrays. These criteria were also true for the antibodies on the targeted array. These two ‘random arrays’, covering 760 antibodies were corresponding to 711 unique proteins, generating the total sum of 1,132 antibodies representing 1,015 unique proteins.

The technical reproducibility of the assays was assessed in independent experiments (shown for ‘targeted array’ in Fig. S1A, S1B and Methods S1 in Text S1). To provide further evidence for the validity of the protein profiles, antibodies raised towards different regions of a common protein were compared (Fig. S7 and Table S3 in Text S1). Principal component analysis (PCA) was chosen as a first tool to visualize a separation between control and disease groups globally. The first three principal components indicated a larger spread separation between the samples analyzed by proteins represented on the ‘targeted array’ compared to the ‘random arrays’ (Fig. S2A in Text S1).

### Identification of single protein candidates differentiating malaria disease groups and healthy parasite-negative community controls

**Figure 2:**
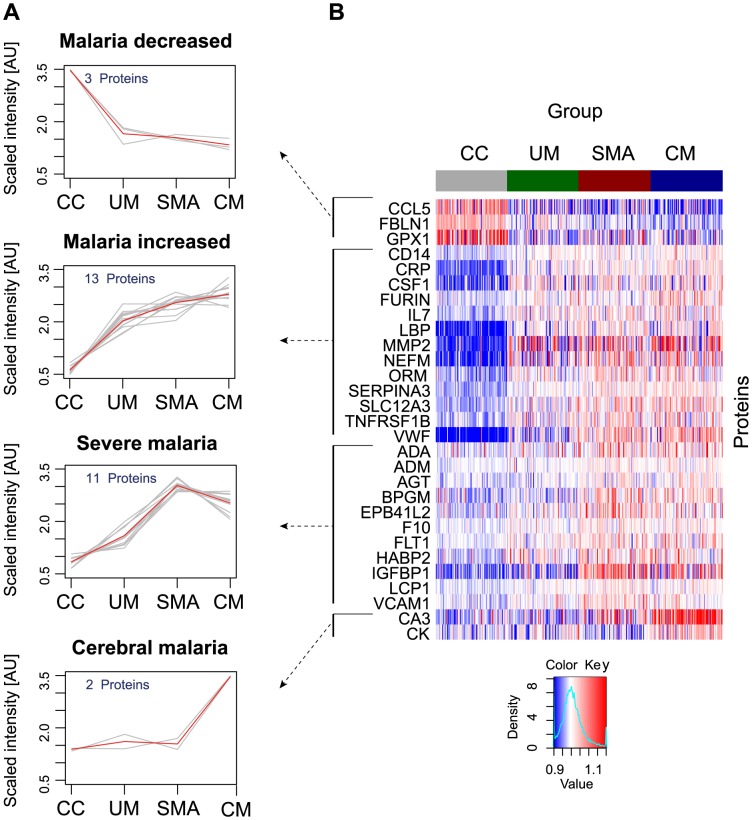
Identification of 29 human proteins discriminating community controls and malaria cases. (**A**) Applying a non-parametric test, 29 human proteins were identified showing significant (adjusted p<0.001) differences between any of the four groups. Cluster analysis using self-organizing tree algorithm (SOTA) revealed four different clusters with distinct protein profiles designated as ‘malaria decreased’, ‘malaria increased’, ‘severe malaria’ and ‘cerebral malaria’ (see also Fig. S3 in Text S1). (**B**) Heatmap visualizing protein profiles in individual patients. Samples were organized according to group affiliation and proteins were sorted following SOTA clusters. Displayed are scaled relative intensities of each protein in each group (CC = grey, UM = green, SMA = red, CM = blue).

A non-parametric test was used to identify proteins that were significantly different (p<0.001) between any of the groups, i.e. UM, SMA and CM as well as healthy parasite-negative CC. From the ‘targeted array’, 29 human proteins were identified in plasma as discriminatory protein targets (Table 2, Fig. 2, Figs. S2B, S3 in Text S1).

**Table 2:** Overview of single protein candidates and SOTA clusters (targeted array).

Cluster	Protein	Uniprot	Gene description	Antibodies	References
**Malaria decreased**	CCL5	P13501	chemokine (C-C motif) ligand 5	HPA010552	[33], [41]
	FBLN1	P23142	fibulin 1	HPA001613	N/A
	GPX1	P07203	glutathione peroxidase 1	HPA044758	[42], [43]
**Malaria increased**	CD14	P08571	CD14 molecule	HPA002127	[44]
	CRP	P02741	C-reactive protein, pentraxin-related	HPA027396	[27], [45]
	CSF1	P09603	colony stimulating factor 1 (macrophage)	HPA022244	N/A
	FURIN	P09958	furin	HPA005905	N/A
	IL7	P13232	interleukin 7	HPA019590	N/A
	LBP	P18428	lipopolysaccharide binding protein	HPA001508	[46]
	MMP2	P08253	matrix metallopeptidase 2	HPA001939	N/A
	NEFM	P07197	neurofilament, medium polypeptide	HPA022845	N/A
	ORM	P02763	orosomucoid 1	HPA047725	[4]
	SERPINA3	P01011	serpin peptidase inhibitor, member 3	HPA000893	[13], [47]
	SLC12A3	P55017	solute carrier family 12, member 3	HPA028748	N/A
	TNFRSF1B	P20333	tumor necrosis factor receptor superfamily, member 1B	HPA004796	N/A
	VWF	P04275	von Willebrand factor	HPA002082	[21], [26], [48]
**Severe Malaria**	ADA	P00813	adenosine deaminase	HPA001399	N/A
	ADM	P35318	adrenomedullin	HPA031806	N/A
	AGT	P01019	angiotensinogen	HPA001557	[49]
	BPGM	P07738	2,3-bisphosphoglycerate mutase	HPA016493	N/A
	EPB41L2	O43491	erythrocyte membrane protein band 4.1-like 2	HPA006642	[50], [51]
	F10	P00742	coagulation factor X	HPA030629	[52]
	FLT1	P17948	fms-related tyrosine kinase 1	HPA011740	[21], [53]
	HABP2	Q14520	hyaluronan binding protein 2	HPA019518	N/A
	IGFBP1	P08833	insulin-like growth factor binding protein 1	HPA046972	N/A
	LCP1	P13796	lymphocyte cytosolic protein 1	HPA019493	N/A
	VCAM1	P19320	vascular cell adhesion molecule 1	HPA034796	[54]
**Cerebral malaria**	CA3	P07451	carbonic anhydrase III, muscle specific	HPA021775	N/A
	CK	P12277	creatine kinase	HPA001254	[55], [56]

N/A: not applicable, no previous known link with malaria.

By using self-organizing tree algorithm (SOTA) cluster analysis on candidate proteins from the ‘targeted array’, four clusters grouped proteins according to disease severity (Fig. 2A). A heatmap built on SOTA clusters demonstrated that all but three proteins were elevated in malaria disease groups compared to controls (Fig. 2B, Fig. S2C in Text S1). Proteins that differed in abundance in plasma between the three syndromes were further visualized in volcano plots as two-group comparisons (Fig. S5 in Text S1).

A total of 17 proteins were found to discriminate malaria disease from controls. The set of proteins that was denoted ‘Malaria decreased’ (Fig. 2) contained fibulin-1 (FBLN1) and RANTES (CCL5), both involved in endothelial cell death, whilst glutathione peroxidase (GPX1) protects from oxidative stress. Decreased levels of these proteins may therefore imply endothelial survival and oxidative stress.

The 14 proteins denoted ‘Malaria increased’ were primarily inflammatory acute phase proteins and increased with severity of the disease. Notably, the most significant candidates differing between controls and malaria groups were major inflammatory components von Willebrand factor (VWF) and C-reactive protein (CRP). Additionally, lipopolysaccharide binding protein (LBP), serpin peptidase inhibitor, member 3 (SERPINA3) and orosomucoid (ORM) are known members of the malaria-induced acute inflammatory response (Fig. S9 in Text S1), while neurofilament-M (NF-M) is an intracellular protein not secreted under healthy conditions, but its increased levels may point towards increased tissue lysis.

A remarkable group of markers were proteins found to be common for ‘severe malaria’ (Fig. 2), defined here as common for CM and SMA. Many of these eleven proteins are general inflammatory proteins. Some have specific roles, such as endothelial cell activation (e.g. VCAM1) and coagulation (factor X). In addition, molecules linked to glucose metabolism, as well as anemia (EMBP4.1-L2), were found in this group of proteins.

Interestingly, two proteins were increased exclusively in plasma of children with CM: carbonic anhydrase III (CA3), which has been reported to be strictly tissue specific and present at high levels in skeletal muscle and creatine kinase (CK), which is involved in energy homeostasis and specific to brain, muscle and heart tissues. These two candidates are associated to muscle tissue and indicate a linkage between muscle damage and CM.

Overall for the ‘discovery cohort’, pathway analysis using the Ingenuity Pathway analysis software (Ingenuity Systems) outlined acute phase signaling as the most significant pathway affected in malaria disease (Fig. S9 in Text S1).

From the analysis using the ‘random array’, 12 proteins revealed significant differences using a non-parametric test (p<0.001, Table 3, Fig. S4 in Text S1). In summary, an additional major inflammatory protein, CCAAT/Enhancer Binding protein-alpha (CEBPA) and three intracellular proteins were found with increased levels in plasma from all malaria-positive groups. In the severe malaria groups, intracellular proteins (TIPIN, MSRB1), components of glucose metabolism (ADSSL1, DNPEP1) and an inflammatory protein (DAPK1) had increased levels, further confirming observations made above with the ‘targeted array’. Moreover, myosin 15A (MYO15A) was increased in the CM group similarly to the ‘cerebral malaria’ cluster components of the ‘targeted array’.

**Table 3:** Overview of single protein candidates with SOTA clusters (random array).

Cluster	Protein	Uniprot	Gene description	Antibodies
**Malaria increased**	ADSSL1	Q8N142	adenylosuccinate synthase like 1	HPA052621
	CCDC102A	Q96A19	coiled-coil domain containing 102A	HPA040598
	EEF2	P13639	eukaryotic translation elongation factor 2	HPA040534
	FAM71F2	Q6NXP2	family with sequence similarity 71, member F2	HPA052240
	TIPIN	Q9BVW5	TIMELESS interacting protein	HPA039704
**Severe Malaria**	CEBPA	P49715	CCAAT/enhancer binding protein (C/EBP), alpha	HPA052734
	DAPK1	P53355	death-associated protein kinase 1	HPA040472
	DNPEP	Q9ULA0	aspartyl aminopeptidase	HPA036398
	HAP1	P54257	huntingtin-associated protein 1	HPA053019
	MSRB1	Q9NZV6	methionine sulfoxide reductase B1	HPA052662
	SEC24C	P53992	SEC24 family	HPA040196
**Cerebral malaria**	MYO15A	Q9UKN7	myosin XVA	HPA039770

In summary, both targeted and blinded selections revealed differential protein profiles in plasma that highlighted several processes in response to the infection and encouraged the subsequent analysis for a classification of malaria disease subtypes.

### Multivariate discrimination of malaria disease subtypes

The list of candidate proteins discriminating the three disease groups contained a number of interesting targets (Table 2, Table S1 in Text S1). However, a multi-protein signature consisting of an optimal combination of these or additional markers generally achieves a more efficient and robust discrimination. Therefore, we chose a L_1_ penalized regression model [18], [19] to identify panels of proteins that would distinguish between each sub-group comparison, as presented below.

**Figure 3:**
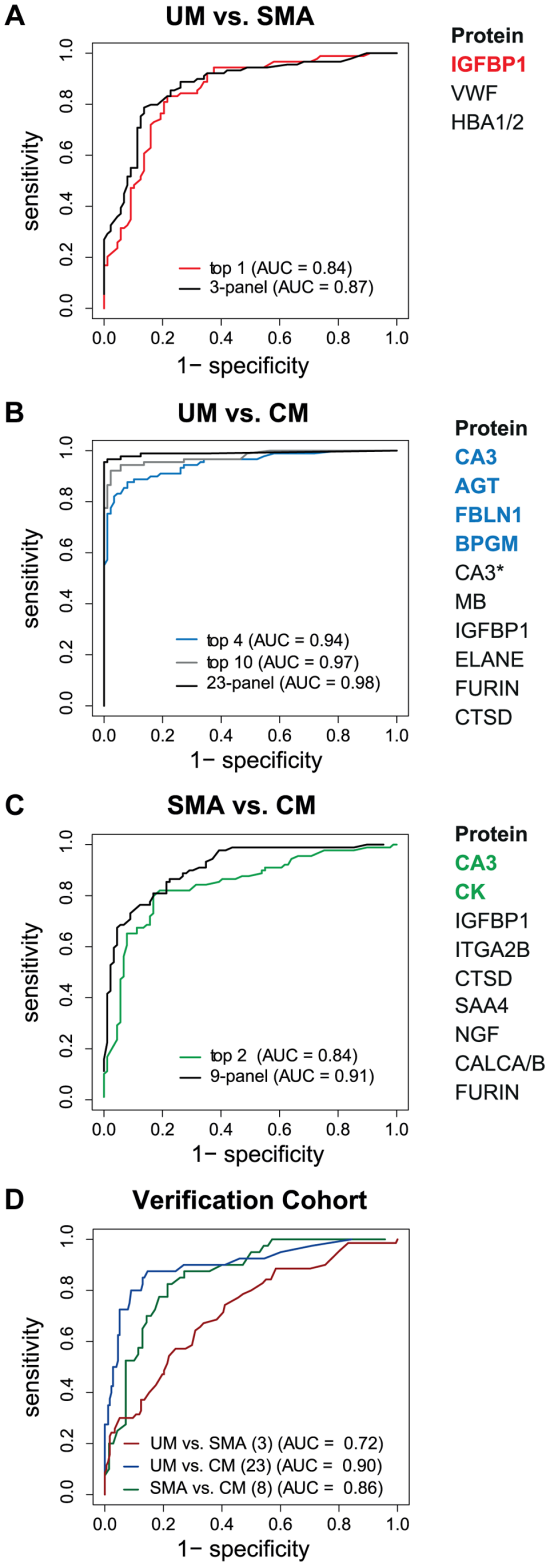
Discrimination of the three malaria disease sub-types with multi-protein signatures. L1-penalized logistic regression models were fitted for the three two-group comparisons. The plots show the resulting ROC curves when the model included all selected proteins (black line) and only the top ones (coloured line). The area under the ROC curve (AUC) for the optimal number of proteins and the combination with the smallest number of proteins after variable selection refinement is presented adjacent to the plots. (**A**) For classification of UM vs SMA a 3-protein signature provided an optimal result (AUC = 0.87) (black line). (**B**) For classification of UM vs CM a protein signature with 23 proteins showed the best result (black line). As comparison, the AUC of the top 4 proteins (blue line) and top 10 proteins (grey line) after step-by-step removal of selected proteins is shown. CA3 = HPA021775, CA3* = HPA026700. (**C**) For classification of SMA vs CM a protein signature with 9 proteins showed the best result (black line). As comparison, the AUC of the top 2 proteins after step-by-step removal of selected proteins is shown (green line). (**D**) ROC curves for the three subgroup comparisons using their respective best protein signatures in the verification cohort, UM vs SMA (red line), UM vs CM (blue line) and SMA vs CM (green line). SAA4 was excluded from verification due to technical failure.

The best predictor protein to differentiate SMA from UM was insulin-like growth factor binding protein 1 (IGFBP1) with an area under the ROC curve (AUC) of 0.84 alone (Fig. 3A, Table S2 in Text S1). The best multi-protein combination was a 3-protein signature consisting of IGFBP1 with von Willebrand factor (VWF) and hemoglobin α-subunit (HBA2, HBA1), which resulted in a slightly improved AUC of 0.87.

Next, Carbonic anhydrase III (CA3) alone discriminated CM from UM with a high AUC of 0.90 (Table S2 in Text S1). Variable selection refinement showed that a panel of 4 proteins, including angiotensinogen (AGT), FBLN1 and 2,3-bisphosphoglycerate mutase (BPGM), resulted in a yet improved performance of 0.94 (Fig. 3B). The optimal signature identified for the discrimination of CM and UM was a 23-protein signature with an AUC of 0.98.

The two best classifier proteins for the comparison of the two severe malaria groups, CM and SMA, were CA3 and CK. The combination of the 2 proteins resulted in an AUC of 0.84 (Fig. 3C). Moreover, the most optimal classifier combination comprised 9 proteins resulting in an AUC of 0.91.

In summary, protein targets in the multivariate signatures overlapped largely with the targets found in the univariate analysis in the two-group comparisons (Fig. S5 in Text S1). As expected, ranking of the proteins differed, because multivariate models aim at the identification of the best combination of proteins to maximize the discriminative power. This resulted in protein signatures that contained not only highly significant proteins found with univariate analysis but proteins that carry important discriminatory information when combined with others.

### Verification of multi-protein panels with independent patient cohorts

Both the single protein classifiers and multi-protein panels were validated with a new and independent set of 363 samples from the same hospital. Using the classifiers determined in the multivariate signatures containing the full list proteins, the AUC for the UM versus SMA (3 proteins), UM versus CM (23 proteins) and SMA versus CM (8 proteins) comparisons were 0.72, 0.90 and, 0.86, respectively (Fig. 3D).

To further verify the results technically, additional antibodies were used targeting other regions of the same protein. For example, von Willebrand factor (VWF) was repeated using the same antibody (HPA00282) and an additional antibody (HPA001815), with both showing comparable results in the different disease groups in the verification cohort and the discovery cohort (Fig. S7 in Text S1), demonstrating reproducibility of the presented results.

Additionally, a small cohort of disease control (DC) samples (Table 1b) from patients suffering from coma or meningitis was also profiled with the same protein panel analyzed in the verification cohort. Changes in levels due to inflammation, as shown with CEBPA, CRP and CCL5 protein levels, were more exacerbated in the CM than the DC samples (Fig. S10 in Text S1) suggesting a stronger inflammatory response in malaria-infected patients than in other diseases.

### Muscle proteins as indicators of cerebral malaria

CA3 was identified as the top candidate to differentiate CM from the other two malaria syndromes. In both the discovery and the verification analysis, two antibodies with distinct specificities to CA3 showed concordant performance (Fig. S7 in Text S1). An additional antibody was acquired against the same target protein and further confirmed the results. Immunohistochemical staining of healthy human tissue confirmed the muscle specificity of both antibodies (Fig. S8 in Text S1). Similarly, antibodies against creatine kinase (CK) revealed increased levels in CM in the discovery phase. The CK antibody used in the discovery phase was raised against a region of the brain-specific form (CKB) that is shared with the muscle-specific (CKM) isoform (Table S3 in Text S1). To further evaluate which isoform is detected, an HPA antibody directed towards the muscle-specific isoform (CKM) as well as a commercially available antibody against CKM were tested in the verification phase. The results from these CKM antibodies were similar to the trends with the CKB/M antibody used in the discovery phase (Fig. S7 in Text S1). Furthermore, immunohistochemical staining showed that the CKB/M antibody recognized skeletal and cardiac muscle as well as cerebral tissues (Fig. S8 in Text S1). Finally, myoglobin (MB), a cardiac and skeletal muscle protein, was also part of the discriminatory profile between CM and UM (Fig. 3b) and also had higher plasma levels in the CM group compared to all other groups in the verification sample set.

Using the small additional DC cohort, CA3 and CK, previously identified as related to CM syndrome, had levels slightly lower in the DC group compared to CM but were not significantly different (Fig. S10 in Text S1) suggesting that muscle damage might not be specific to cerebral malaria but probably linked to coma. Further studies with a larger cohort of DC comatose group of children with pathologies other than malaria are required to verify these findings.

## Discussion

We have here investigated the levels of human proteins circulating in plasma of children with different forms of uncomplicated or severe malaria and compared the levels with those of parasite-negative community controls. The study comprises a total of 709 plasma samples including 515 from malaria-infected children. Amongst the 1,015 host proteins studied, 41 were identified as candidates discriminating between healthy community controls and malaria patients. Protein markers of oxidative stress were found elevated in anemic individuals while markers of endothelial activation, platelet adhesion and muscle- and tissue damage were found linked to cerebral malaria. Taken together, this suggests the presence of a generalized vascular inflammation, an unbalanced glucose metabolism and deep lesions into the micro-vasculature.

The chosen bead array technology enabled the generation of protein profiles in unfractionated and biotinylated plasma samples by using combinations of large sets of antibodies as demonstrated by the use of both carefully pre-selected and blindly chosen antibodies.

Most previous studies have focused on markers of fatalities in those already with severe malaria [20]–[22]. In one recent analysis [14], discrimination of different malaria syndromes from each other was suggested possible but only when using extensive protein panels with up to 50 proteins (AUC of 0.7–0.8). In contrast, we show herein that, employing small panels of proteins, it is possible to build models that predict with an AUC higher than 0.90 which children have severe malaria complications. We also demonstrate a discriminatory signature that reaches superior accuracy for UM vs. SMA with IGFBP1 alone and for UM vs. CM using only four proteins.

Higher plasma levels of muscle-derived proteins were found in children with cerebral malaria only including carbonic anhydrase III and creatine kinase, suggesting that smooth muscle-cells of the microvasculature may be injured. The excessive sequestration of pRBC seen in cerebral vessels, the level of which has also been found to correlate with coma [3] is probably one of the reasons for the injury of the muscle cells. This is in concordance with previous histo-pathological, studies where subjects who succumbed to cerebral malaria showed vascular- and microvascular lesions complicated by ring-hemorrhages [3]. The presence of myoglobin in the plasma of the patients with CM, a marker of cardiac- and striated muscles only, also indicates that the vasculature and the muscles outside of the brain are severely affected by sequestration as seen for example in muscle biopsies of Thai adults [23]. Further, a recent study showed that blood flow obstruction might be exacerbated by increased skeletal muscle oxygen consumption in severe malaria [24], contributing to hypoxic and hyperlactemic conditions in the microvasculature. Lack of oxygen in muscle cells accompanied with hypoglycemia, lactate overproduction, oxidative stress and inflammation are typical consequences of muscle damage [25], which could further contribute to vascular injuries and subsequent muscle cell death with the release of muscle-specific proteins into the blood circulation. Whether creatine kinase, myoglobin and carbonic anhydrase III release in the plasma exacerbate these deleterious events is not known at this stage and deserves further investigation. For example, increased plasma carbonic anhydrase activity could contribute to the impairment of the acid-base and excess myoglobin in blood circulation could lead to kidney failure if filtrated by kidneys. An additional small cohort of malaria-negative children, with other illnesses involving coma, suggested that coma could either be a cause or a consequence of muscle damage observed in cerebral malaria, similarly to other comatose diseases. Further studies, involving larger cohorts of non-malaria comatose children, will be required to verify this hypothesis.

**Figure 4:**
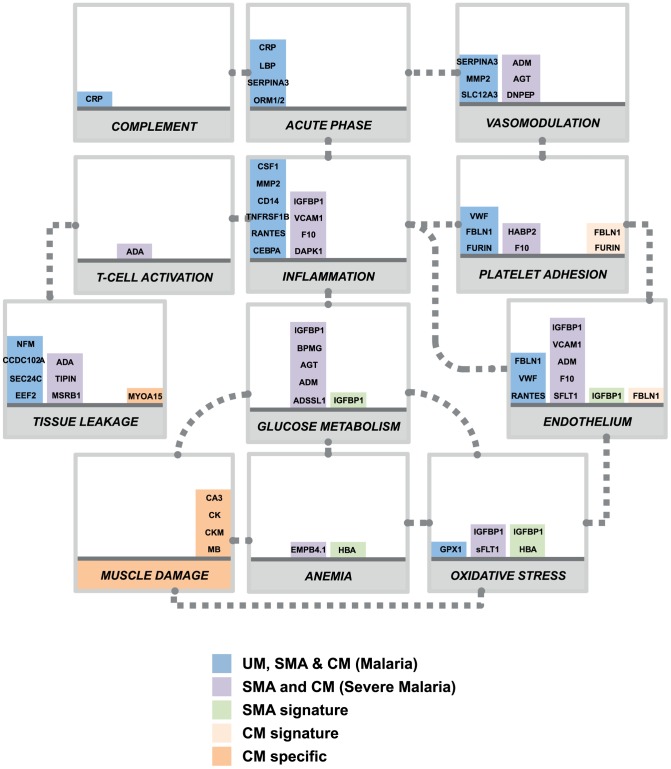
Overview of panels of proteins and their physiological pathways predicting the different malaria clusters. Proteins with differential profiles between the malaria groups were classified into physiological pathways. This included proteins identified common to all malaria groups (blue bars), common to both defined severe malaria syndromes (purple bars), proteins with levels elevated in SMA (light green bars), proteins with levels elevated in CM (light orange bars) and proteins specific to CM (dark orange bars). For each panel, columns were stacked by number of proteins identified in each category. Grey dotted connectors represent either a regulation link or a common protein component between to physiological pathways. UM: uncomplicated malaria; SMA: severe malaria anemia; CM: cerebral malaria. Proteins are represented by their gene names (Refer to Table 2, Fig. S3 and Table S2 in Text S1 for full names).

In our study, predicting which samples were from patients diagnosed with cerebral malaria was very accurate in both cohorts due to the discovery of the presence of the muscle-proteins only in the plasma of the children with cerebral malaria. Previous studies have successfully used endothelial cell activation markers to predict severe malaria, notably using angiopoietin-1 and 2 [20], [21], [26], but their specificity to cerebral malaria, as compared to other severe complications, remains unclear. Here we propose that markers of muscle damage accompanied by markers of endothelial cell activation/platelet adhesion in the plasma (Fig. 4, orange bars) are specific to cerebral malaria pathogenesis and distinct to severe malaria anemia. Our data therefore indicate that children with uncomplicated malaria that develop cerebral malaria are likely to have vascular lesions and muscle damage, which can be readily monitored in plasma.

Most of the plasma proteins showing differences in between the malaria patients and the community controls were components of the inflammatory response (Fig. 4, blue bars). Further, multiple novel inflammatory components were identified, including MMP2, CSF1, and IL7 (table 2), demonstrating the presence of a more generalized vascular inflammation in patients with malaria infections. The reliability of the present study was furthermore confirmed by the fact that a number of protein measured herein include acute phase- and inflammatory proteins previously documented to be present in the plasma of malaria infected individuals [27], [28] (Table 2). Proteins related to different aspects of the glucose metabolism, including insulin-glucagon modulators and glycolytic enzymes, were also elevated in both SMA and CM patients compared to those with UM (Fig. 4, purple bars). Further investigation of their potential role in the induction of hypoglycemia, a hallmark of severe malaria associated with fatality [29], [30] might refine knowledge on the human response mechanisms to malaria infection.

Most proteins elevated in plasma from SMA compared to UM were also elevated in CM but to a lower extent. Yet, IGFBP1 and HBA were part of the protein signature for comparing SMA with UM, and had highest levels in patients suffering with SMA. IGFBP1 could be further expressed due to high levels of reactive species [31], and free-hemoglobin release in blood circulation could be a trigger of free-heme induced oxidative stress, particularly if not properly scavenged by the haptoglobin-hemopexin system [32]. It is noteworthy that predictive protein signatures for SMA have in previous studies mainly included inflammatory cytokines [22], [33], [34]. For example, we recently showed in the same Nigerian population, that pro-inflammatory cytokines were more pronounced in SMA than in CM [9], a finding supported by the fact that pro-inflammatory TNF-alpha has a role in anemia establishment [8]. Due to the sensitivity of the assay, the levels of most of the cytokines tested in the present study were too low to be detected. Consequently, we hypothesize that including oxidative stress-related proteins as well as pro-inflammatory cytokines in future studies in the protein signature could potentially assist to further improve SMA distinction from other malaria complications.

In summary, a high-throughput antibody-based protein profiling method and large-scale discovery and verification cohorts, revealed muscle-specific proteins in plasma as potential indicators of cerebral malaria. Our study could therefore provide key elements towards the discovery of distinct mechanisms in the human response to malaria infection between the two most fatal syndromes of childhood malaria.

## Materials and Methods

### Ethics statement

Parents or guardians of study participants gave informed written consent. This research was approved by the internationally accredited joint ethics committee of the College of Medicine of the University of Ibadan and the University College Hospital, Ibadan.

### Study design

All study participants with illness were recruited under the auspices of the Childhood Malaria Research Group (CMRG) at the University College Hospital (UCH) in the city of Ibadan, Nigeria. Malaria-negative community control (CC) children were recruited from local vaccination clinics as well as during school visits across several Ibadan districts. This case-control study was divided in a discovery cohort that contains those patients recruited during 2006 to 2011 and a verification cohort made up of those recruited in the 2009 to 2012 period.

Children were aged from 6 months to 13 years and were screened for parasite detection by microscopy following Giemsa staining of thick and thin blood films as performed routinely at UCH. Clinical definitions used were as defined by the WHO criteria for severe *P. falciparum* malaria [1]. Uncomplicated malaria (UM) cases were defined as febrile children with *P. falciparum* parasitaemia and PCV (Packed Cell Volume) greater than 20% who did not require hospital admission. Severe malarial anemia (SMA) cases were defined as conscious children with PCVs less than 16% in the presence of *P. falciparum* parasitaemia. Cerebral malaria (CM) cases were defined as children in unrousable coma for at least one hour in the presence of asexual *P. falciparum* parasitaemia with normal cerebrospinal fluid and PCV greater than 20%. Community controls were children that did not show any obvious symptoms of illness and seemed healthy. They were screened for parasite presence and were only included in the study if the Giemsa staining of both thick and thin blood films were negative for *Plasmodium* parasites. They were selected to match age and sex with malaria-infected patients. The clinical data was compiled for each patient and samples were collected as previously described [9], [14] (see Methods S1 in Text S1).

### Antibody selection and array design

Antibodies were selected and acquired from the huge antibody collection within the Human Protein Atlas (HPA, www.proteinatlas.org) consisting of more than 40,000 antigen purified and protein microarray validated antibodies.

The selection of antibodies was carried out using two different strategies. Using a ‘targeted’ approach, 380 antibodies were selected against 304 protein targets according to a generous and inclusive literature mining of malaria pathogenesis. The final set was defined by availability and fulfillment of technical validations, such as having a concentration that is higher than 50 µg/ml and that the specificity is validated on planar protein arrays. The two additional sets of 380 antibodies were randomly chosen from the routine antibody production within HPA. These 760 antibodies were directed to 711 unique proteins and fulfilled the same criteria as for the antibodies on the targeted array.

### Suspension bead array procedure

For the generation of antibodies suspension beads, antibodies were diluted using a liquid handling system (EVO150, Tecan) and coupled to carboxylated magnetic microspheres (MagPlex, Luminex Corporation) as previously described [35]. Briefly, carboxylated beads were activated with 1-ethyl-3-(3-dimethylaminopropyl) carbodiimide (EDC) and *N*-hydroxysulfosuccinimide (Sulfo-NHS, Thermo Scientific) and incubated with 1.6 µg antibody in a multi-well microtiter plate for 2 h. After the coupling reaction, beads were stored at 4°C in a protein containing buffer (Blocking Reagent for ELISA, Roche Applied Science) supplemented with ProClin (Sigma-Aldrich). Before incubation with samples, the different bead identities were combined to create a 384-plex-bead array. Antibody coupling was confirmed with R-phycoerythrin (PE)-conjugated donkey anti-rabbit IgG antibody (Jackson ImmunoResearch).

The data from single antibody and direct sample labeling assays was judged by technical replication of the experiment, profile concordance of several antibodies raised towards a common target protein, and biological replication of the analysis in new, independent samples.

Biotinylation of plasma samples was performed as previously described [36] (refer to Methods S1 in Text S1). Biotinylated samples were then diluted in PBS containing 0.5% (w/v) polyvinylalcohol, 0.8% (w/v) polyvinylpyrrolidone, 0.1% casein (all from Sigma) supplemented with 0.5 mg/ml rabbit IgG (Bethyl) using a liquid handler (SELMA, CyBio). Before incubation with bead arrays, samples were heat-treated in a thermocycler for 30 min at 56°C for epitope retrieval. After incubation of samples with beads for 14 h, beads were washed with PBS-T (pH 7.4, 0.05% Tween20) on a plate washer (EL406, Biotek) and incubated with 0.4% paraformaldehyde for 10 min. Subsequently, beads were incubated with 0.5 µg/ml R-Phycoerythrin labeled streptavidin (Invitrogen) for 20 min and washed with PBS-T. Bead identities and median fluorescence intensity of R-Phycoerythrin were analyzed simultaneously using a FlexMAP 3D system (Luminex Corp.).

### Statistical analysis

Data analysis was performed using R language for statistical computing [37], [38]. The data from the ‘targeted array’ and the ‘random array’ was normalized using probabilistic quotient normalization (PQN) as described before [39]. The non-parametric Kruskal-Wallis test was applied to identify proteins that are different among the different malaria disease groups (CC, UM, SMA and CM). For pairwise comparison of the different malaria disease groups, a Wilcoxon rank sum test was applied (with continuity correction). Bonferroni method was used to control the family-wise error rate. For cluster analysis of the protein profiles self-organizing tree algorithm (SOTA) was applied after the medians per protein and subgroup were centered and scaled using the R function scale [40] (Methods S1 in Text S1).

The data from the verification cohort was normalized using a linear mixed model
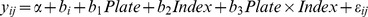
where: y = log2(intensity), Plate: biotinylation plates, Index: order of assay, and *b*_i_: random effect of the *ith* target.

For the identification of protein signatures a logistic regression model was used. We used L_1_ penalization proposed by Tibshirani [19], also known as Lasso, which performs parameter estimation and variable selection at the same time. The penalization involves a penalize parameter (λ) which is chosen through a cross-validation procedure. Thereby, the dataset was randomly divided into subsets. The first subset (K) was designated as the test dataset, while the model was fitted to the remaining training dataset (subset K-1). This procedure was repeated k times for each subset (see Methods S1 in Text S1 for details). For a first verification of the identified multi-protein signatures, the parameter estimates from the first dataset were used to obtain the prediction based on the second replicate data of the discovery cohort (Fig. S6 in Text S1).

## Supporting Information

Text S1 **Supplementary Information.** An extensive set of supplementary material is available with detailed descriptions and visualizations of the data and the identified proteins of interest with ten figures, four tables and methods.
(PDF)
