# Supplementary material for: Affinity Proteomics Reveals Elevated Muscle Proteins in Plasma of Children with Cerebral Malaria
Source: PLoS Pathog. 2014 Apr 17;10(4):e1004038. doi: 10.1371/journal.ppat.1004038 (PMC3990714; doi:10.1371/journal.ppat.1004038)
Supplement: Text S1 — Supplementary Information. An extensive set of supplementary material is available with detailed descriptions and visualizations of the data and the identified proteins of interest with ten figures, four tables and methods. (PDF) [file ppat.1004038.s001.pdf]

Supporting Information

Affinity proteomics reveals elevated muscle proteins in plasma of children with cerebral malaria

Julie Bachmann[1#], Florence Burté[2#], Setia Pramana[3], Ianina Conte[2], Biobele J. Brown[4,5,6], Adebola E. Orimadegun[3], Wasiu A. Ajetunmobi[4], Nathaniel K. Afolabi[4], Francis Akinkunmi[4], Samuel Omokhodion[4,6], Felix O. Akinbami[4,6], Wuraola A. Shokunbi[5,6], Caroline Kampf[7], Yudi Pawitan[3], Mathias Uhlén[1], Olugbemiro Sodeinde[2,4,5,6], Jochen M. Schwenk[1], Mats Wahlgren[8*], Delmiro Fernandez-Reyes[2,4,5,6*], Peter Nilsson[1*]

Legends to Supplementary Information

Figure S1. Intra-assay and inter-assay variance of suspension bead array. A) To analyze the intra-assay variance of one multiplex array, the coefficient of variation (CV) of each antibody (k=384) was determined in sample replicates (n=24) of one array experiment. 350 antibodies showed a CV of <10% and only 2 antibodies were >20%. B) The technical inter-assay variance was evaluated by comparing sample-wise and antibody-wise correlations of two independently performed array experiments. The Spearman correlation coefficient between each duplicated sample was *rho*<0.94. Antibodies in two independent arrays showed a median Spearman *rho*=0.88 ranging between 0.45 and 0.99. In both experiments two samples were excluded due to technical failure. C) Sample- and antibody-wise correlation coefficients for the 24 duplicated patient samples in discovery and verification set with the top 29 proteins.

Figure S2. A) Principal component analysis (PCA) visualizing samples of “random” and “targeted” antibody profiling (k = 384 antibodies, n = 354 samples). B) Principal component analysis (PCA) of samples with the top significant 29 proteins from the “targeted array” (k = 29 antibodies, n = 354 samples). Three-dimensional plots based on the first three dimensions of the PCA results are presented. All samples are colored based on the respective group affiliation. C) Heatmap showing protein profiles of the identified 29 host proteins. Samples were clustered based on Euclidian distance using the complete linkage method for hierarchical clustering. Proteins were sorted according to SOTA clusters. CC = grey, UM = green, SMA = red, CM = blue.

Figure S3. Correlations and boxplots of the 29 identified discriminatory proteins from the ‘targeted array’. The discovery screening using the ‘targeted array’ was performed in two independent experiments. For each of the 29 significant proteins corresponding correlations with the Spearman coefficient (*rho)* and boxplots from experiment 1 (Exp1) and

the replicated experiment 2 (Exp2) are represented. All antibodies with significant differences showed *rho*>0.8.

Figure S4. Boxplots of the identified proteins from the 'random arrays'. Aiming at the identification of new proteins that have not been described in the context of malaria before, a part of the discovery screening was performed using 'random arrays'. These were created using a random selection of 760 antibodies being compiled from the collection of validated antibodies within HPA. Applying a non-parametric test (Kruskal-Wallis), 12 proteins were identified showing significant differences (p<0.001) between at least two of the four groups. ADSSL1 = adenylosuccinate synthase-like 1, CEBPA = CCAAT/enhancer binding protein (C/EBP)/ alpha, FAM71F2 = family with sequence similarity 71/ member F2, MSRB1 = methionine sulfoxide reductase B1, HAP1 = huntingtin-associated protein 1, DAPK1 = death-associated protein kinase 1, DNPEP = aspartyl aminopeptidase, MYO15A = myosin XVA, CCDC102A = coiled-coil domain containing 102A, SEC24C = SEC24 family, EEF2 = eukaryotic translation elongation factor 2, TIPIN = TIMELESS interacting protein.

Figure S5. Single proteins with significant differences between the three malaria disease groups. For pairwise comparisons of the groups, Wilcoxon rank sum test was applied (with continuity correction). A volcano plot displaying the relationship between the fold-change and significance was generated. Proteins with a p-value < 0.1^{-6} (corresponds to Bonferroni adjusted p-value < 0.001) are displayed in red. See Table S1 in Text S1 for further details.

Figure S6. Technical verification of multivariate model and variable selection refinement. A) Area under ROC curve (AUC) of identified combinatory panels using the technical replicate data set in the discovery phase. For a first verification of the identified multi-protein signatures, the parameter estimates from the first dataset were used to obtain the prediction based on the second replicate data of the discovery cohort. B) Variable selection refinement. In order to investigate, which proteins contribute predominantly to the classification performance, the classifiers were sorted based on their absolute parameters obtained from L1-penalized logistic model. The logistic models of top *k* proteins were fitted and the AUC was calculated for each model. The effect on the classification performance of including the proteins is represented by the improvement of the AUC (see also Table S2 in Text S2).

Figure S7. Boxplots of CA3, CK and VWF analyzed with different antibodies in the discovery and the verification set. Various antibodies generated against different antigen sequences were used to validate the protein profile of (A) CA3, (B) CK and (C) VWF in the discovery and in the verification cohort. CA3: Carbonic anhydrase (R&D, #AF2185), CKM:

Creatine kinase-M isoform (R&D, #MAB5564 and #G113C), vWF: von Willebrand Factor. DC = Discovery cohort, VC = Verification cohort.

Figure S8. Immunohistochemical analysis of antibodies using bright field microscopy. A) Protein expression (brown) and counterstaining (blue) of anti-CA3, -CK and -MB antibodies on healthy adult tissues is presented. All 5 antibodies showed cytoplasmic positivity in skeletal muscle. Heart muscle displayed cytoplasmic immunoreactivity with 3 out of 5 antibodies used in the study. One candidate showed negative cytoplasmic staining in CNS. B) Immunohistochemical analysis of anti-VWF and anti-SERPINA3 antibodies. Most tissues showed moderate to strong immunoreactivity in blood vessels. HPA00893 lacked blood vessel positivity in heart muscle. HPA02560 showed weak positivity in heart muscle and lacked immunoreactivity in cerebellum. CK: creatine kinase; CA3: Carbonic anhydrase 3; MB: Myoglobin; VWF: von Willebrand Factor; SERPINA3: serpin peptidase inhibitor, member 3.

Figure S9. Properties of targeted antibody array. Ingenuity Pathway analysis software (Igenuity Systems) was used to identify the most important canonical pathways that were enriched in the selected antibody array compared to the Ingenuity default background. *Acute phase signaling* was enriched most significantly and is displayed. Nearly all plasma proteins that are secreted during *acute phase signaling* were covered in the targeted array (orange circles). In the SOTA cluster 'Malaria increased' (see Fig. 2) were found 6 of 46 acute phase signaling plasma proteins: CRP, LBP, ORM1, SERPINA3, TNFRSF1B and VWF (red circles).

Figure S10: Boxplots of the significant proteins and muscle proteins from the DC cohort. The verification screening using was performed in an additional group to the discovery cohort and directely compared to the CM group. The Boxplots for muscle specific proteins and significant proteins is shown .

Table S1. Univariate two-group comparisons.

Table S2. Plasma protein signatures discriminating malaria disease groups after variable selection refinement.

Table S3. Antibody-antigen properties**.**

Table S4. List of target proteins used in the targeted array

Methods S1. More detailed information regarding the procedures utilized for: Case definitions, Clinical data and sample collection, Biotinylation of plasma samples, Quality assessment of suspension bead-array, Immunohistochemistry, Self-organizing tree algorithm (SOTA) analysis, Multivariate analysis.

Supplementary Figures

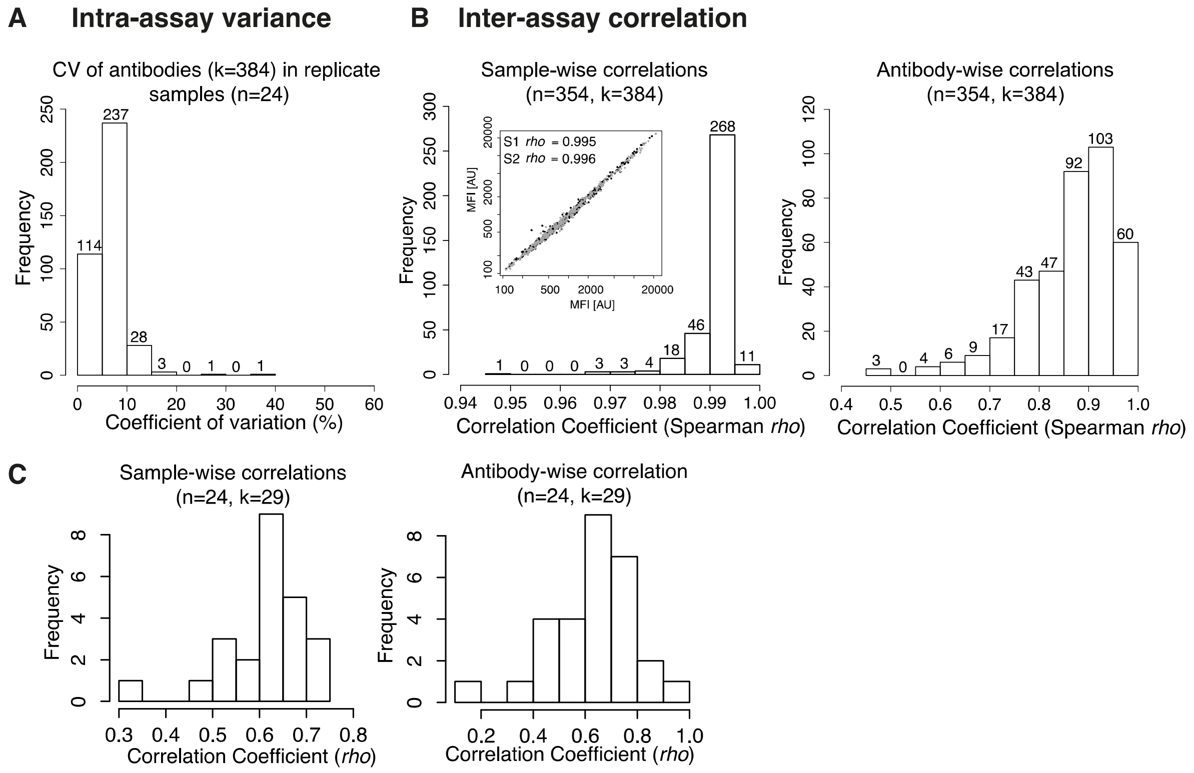

Figure S1.

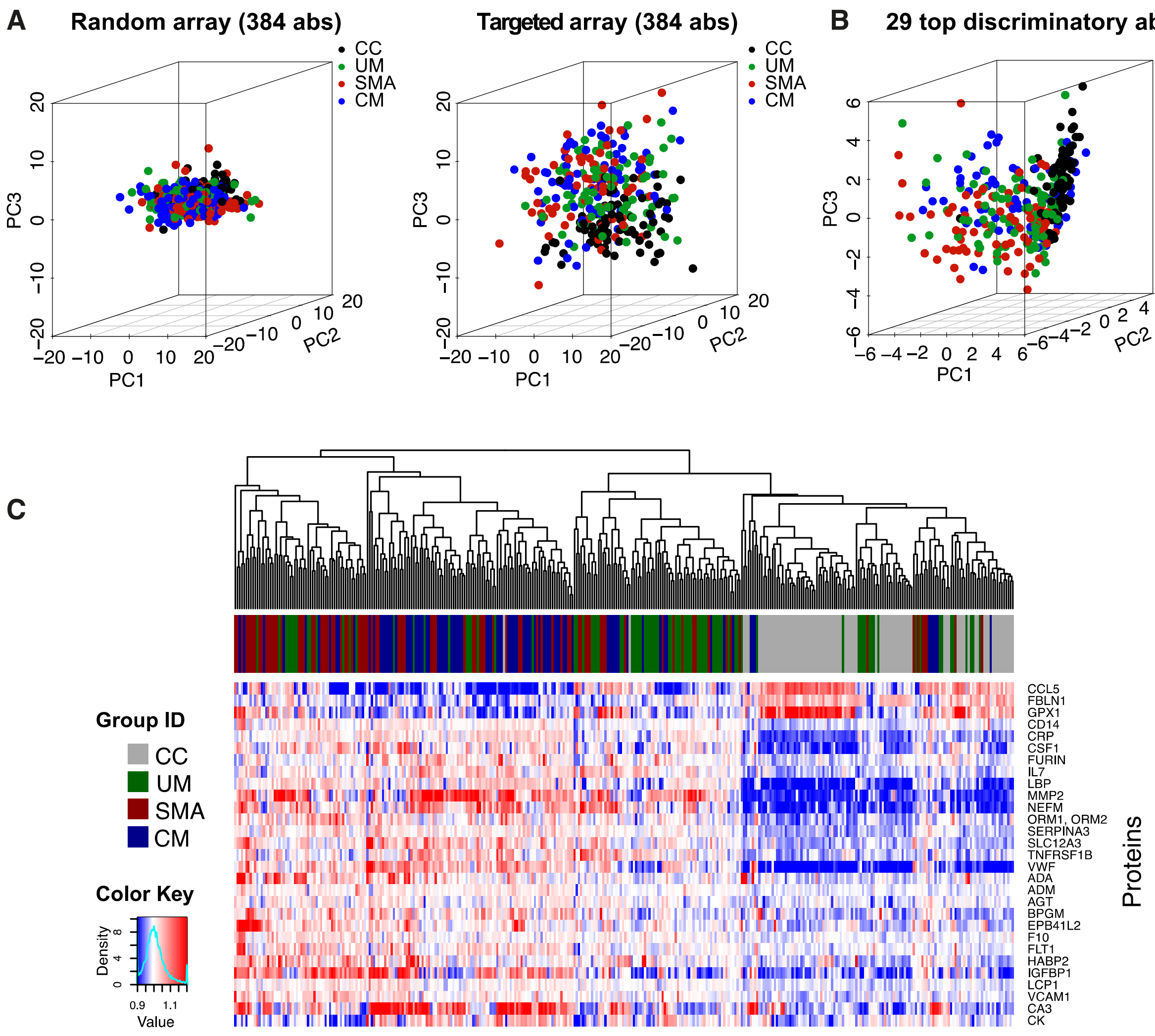
A
Random array (384 abs)
Targeted array (384 abs)
B
29 top discriminatory abs
CC
UM
SMA
CM
PC1
PC2
PC3
C
Group ID
CC
UM
SMA
CM
Color Key
Density
Value
CCL5
FBLN1
GPX1
CD14
CRP
CSF1
FURIN
IL7
LBP
MMP2
NEFM
ORM1, ORM2
SERPINA3
SLC12A3
TNFRSF1B
VWF
ADA
ADM
AGT
BPGM
EPB41L2
F10
FLT1
HABP2
IGFBP1
LCP1
VCAM1
CA3
CK
Proteins

Figure S2.

Figure S3

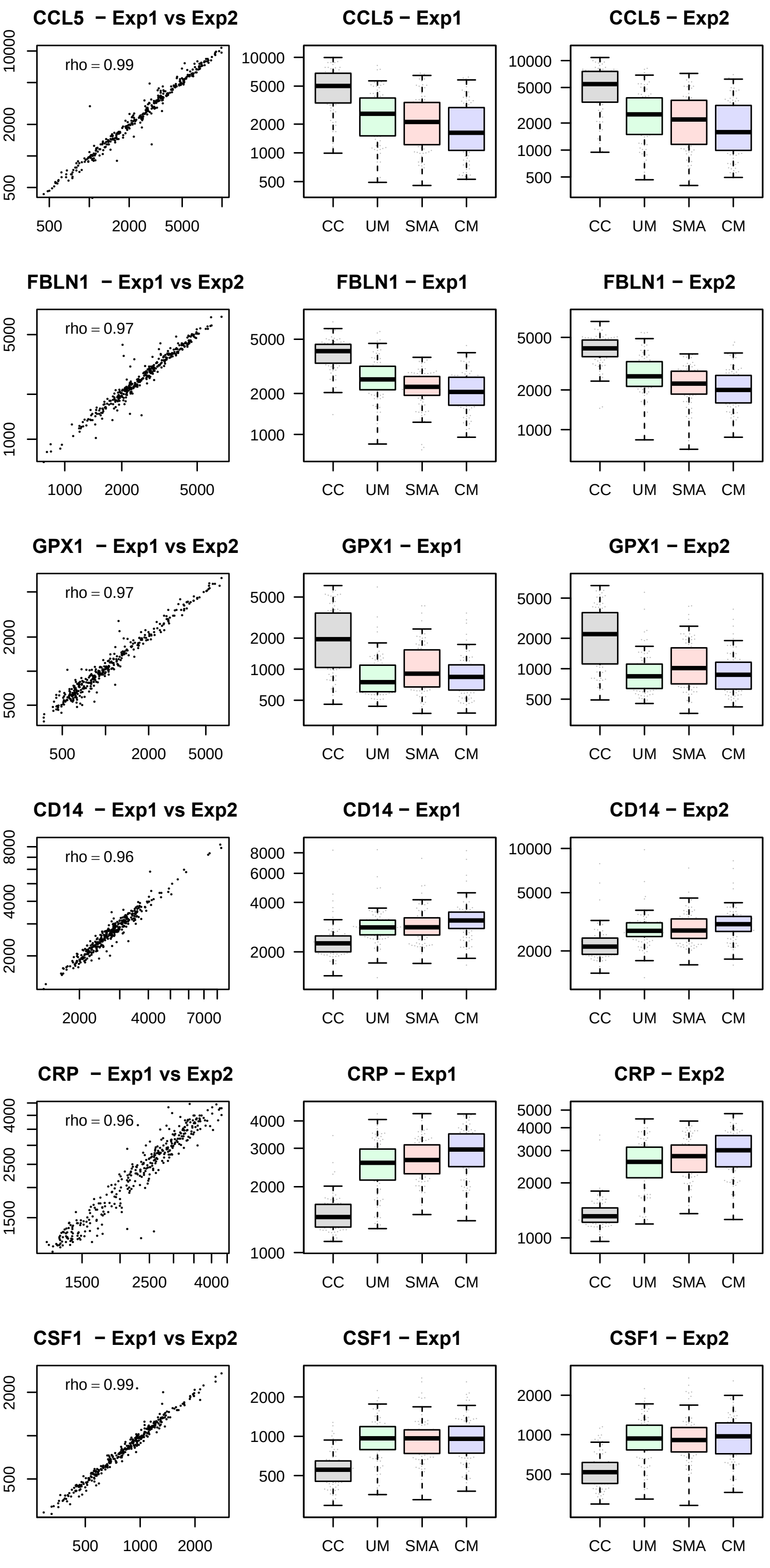
CCL5 – Exp1 vs Exp2
rho = 0.99
CCL5 – Exp1
CCL5 – Exp2
FBLN1 – Exp1 vs Exp2
rho = 0.97
FBLN1 – Exp1
FBLN1 – Exp2
GPX1 – Exp1 vs Exp2
rho = 0.97
GPX1 – Exp1
GPX1 – Exp2
CD14 – Exp1 vs Exp2
rho = 0.96
CD14 – Exp1
CD14 – Exp2
CRP – Exp1 vs Exp2
rho = 0.96
CRP – Exp1
CRP – Exp2
CSF1 – Exp1 vs Exp2
rho = 0.99
CSF1 – Exp1
CSF1 – Exp2
CC
UM
SMA
CM

Figure S3

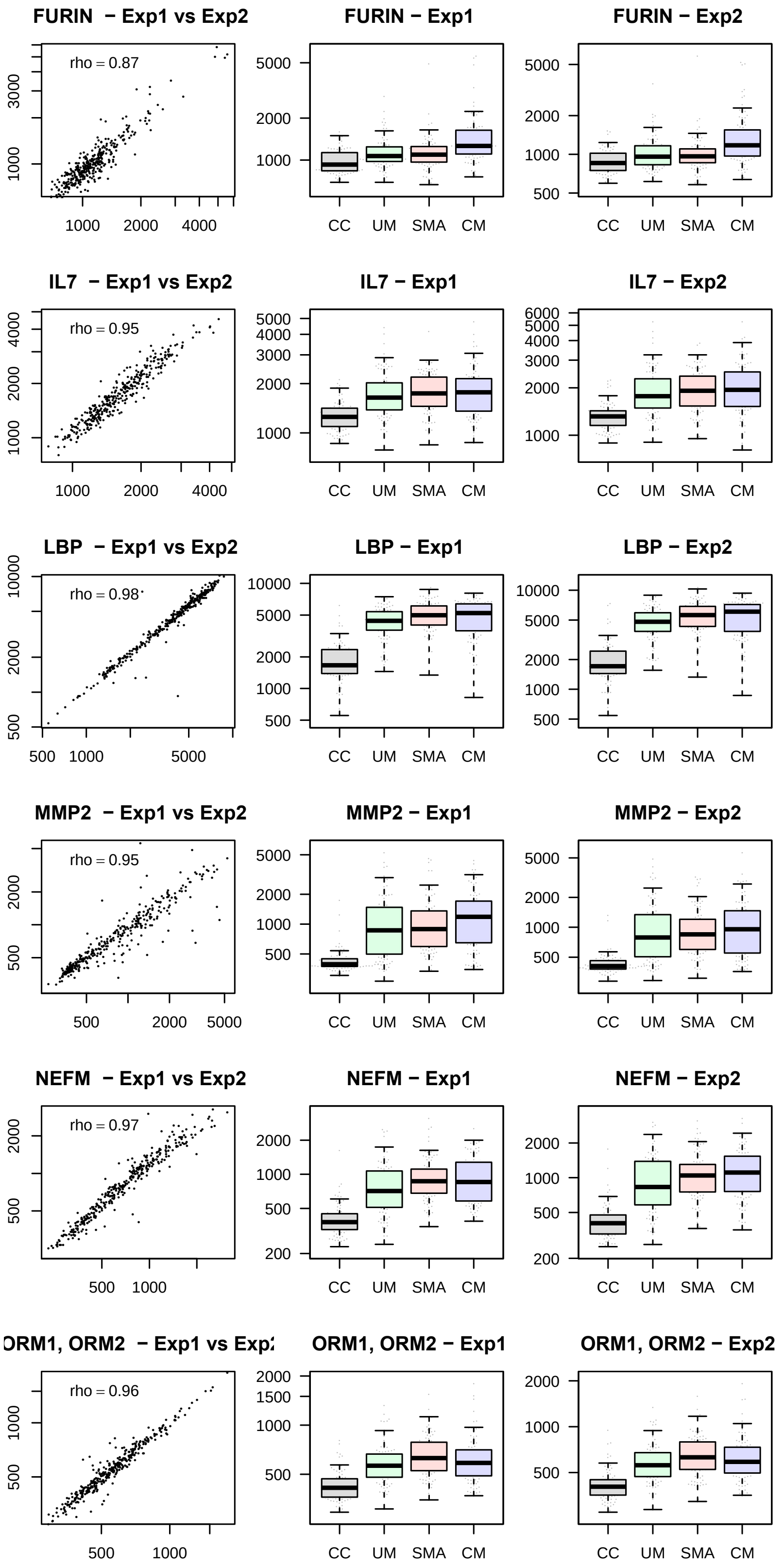

FURIN − Exp1 vs Exp2
rho = 0.87
FURIN − Exp1
FURIN − Exp2
IL7 − Exp1 vs Exp2
rho = 0.95
IL7 − Exp1
IL7 − Exp2
LBP − Exp1 vs Exp2
rho = 0.98
LBP − Exp1
LBP − Exp2
MMP2 − Exp1 vs Exp2
rho = 0.95
MMP2 − Exp1
MMP2 − Exp2
NEFM − Exp1 vs Exp2
rho = 0.97
NEFM − Exp1
NEFM − Exp2
ORM1, ORM2 − Exp1 vs Exp2
rho = 0.96
ORM1, ORM2 − Exp1
ORM1, ORM2 − Exp2
CC
UM
SMA
CM

Figure S3

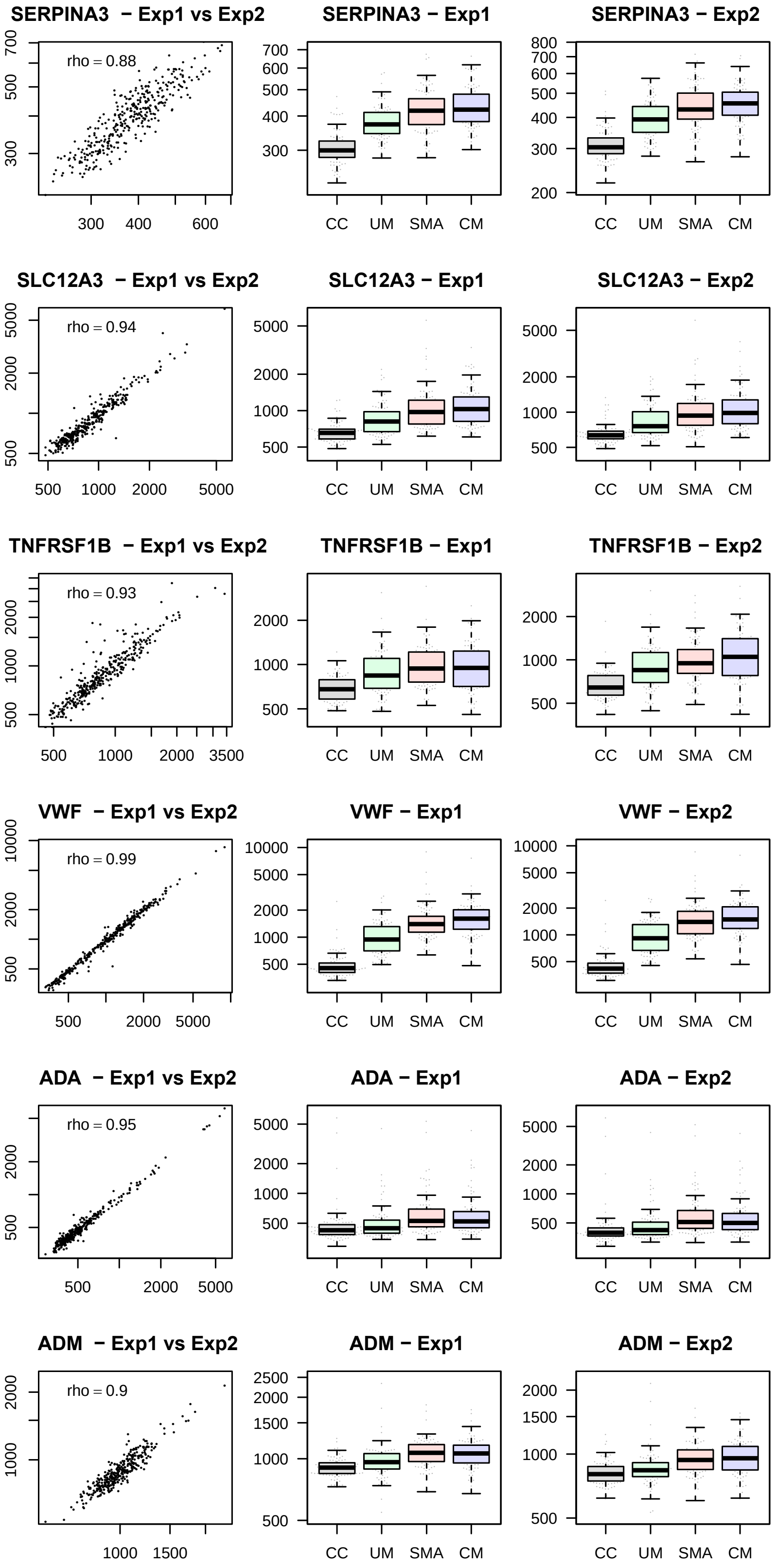
SERPINA3 − Exp1 vs Exp2
rho = 0.88
SERPINA3 − Exp1
SERPINA3 − Exp2
SLC12A3 − Exp1 vs Exp2
rho = 0.94
SLC12A3 − Exp1
SLC12A3 − Exp2
TNFRSF1B − Exp1 vs Exp2
rho = 0.93
TNFRSF1B − Exp1
TNFRSF1B − Exp2
VWF − Exp1 vs Exp2
rho = 0.99
VWF − Exp1
VWF − Exp2
ADA − Exp1 vs Exp2
rho = 0.95
ADA − Exp1
ADA − Exp2
ADM − Exp1 vs Exp2
rho = 0.9
ADM − Exp1
ADM − Exp2
CC
UM
SMA
CM

Figure S3

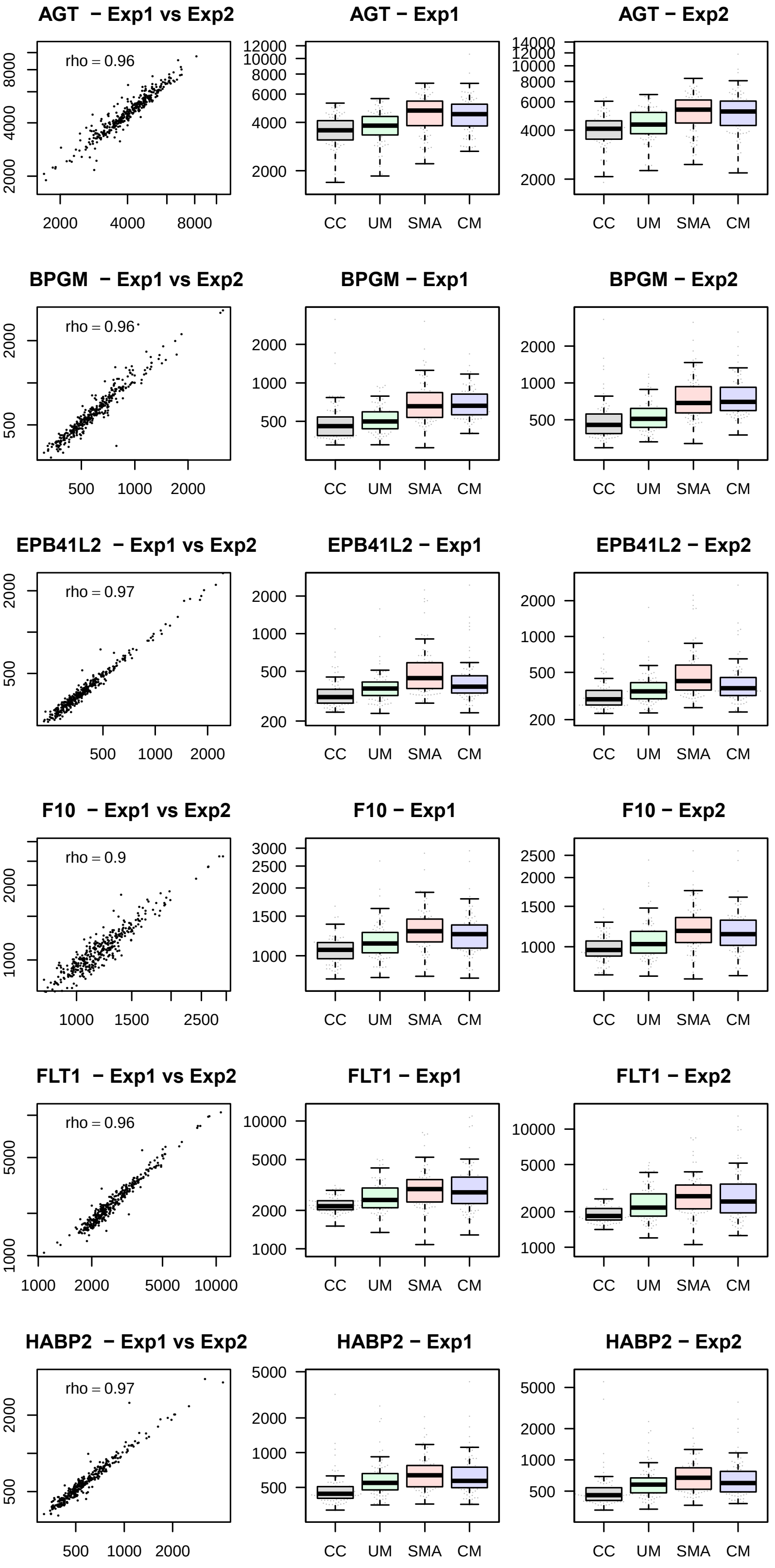
AGT – Exp1 vs Exp2
rho = 0.96
AGT – Exp1
AGT – Exp2
BPGM – Exp1 vs Exp2
rho = 0.96
BPGM – Exp1
BPGM – Exp2
EPB41L2 – Exp1 vs Exp2
rho = 0.97
EPB41L2 – Exp1
EPB41L2 – Exp2
F10 – Exp1 vs Exp2
rho = 0.9
F10 – Exp1
F10 – Exp2
FLT1 – Exp1 vs Exp2
rho = 0.96
FLT1 – Exp1
FLT1 – Exp2
HABP2 – Exp1 vs Exp2
rho = 0.97
HABP2 – Exp1
HABP2 – Exp2
CC UM SMA CM

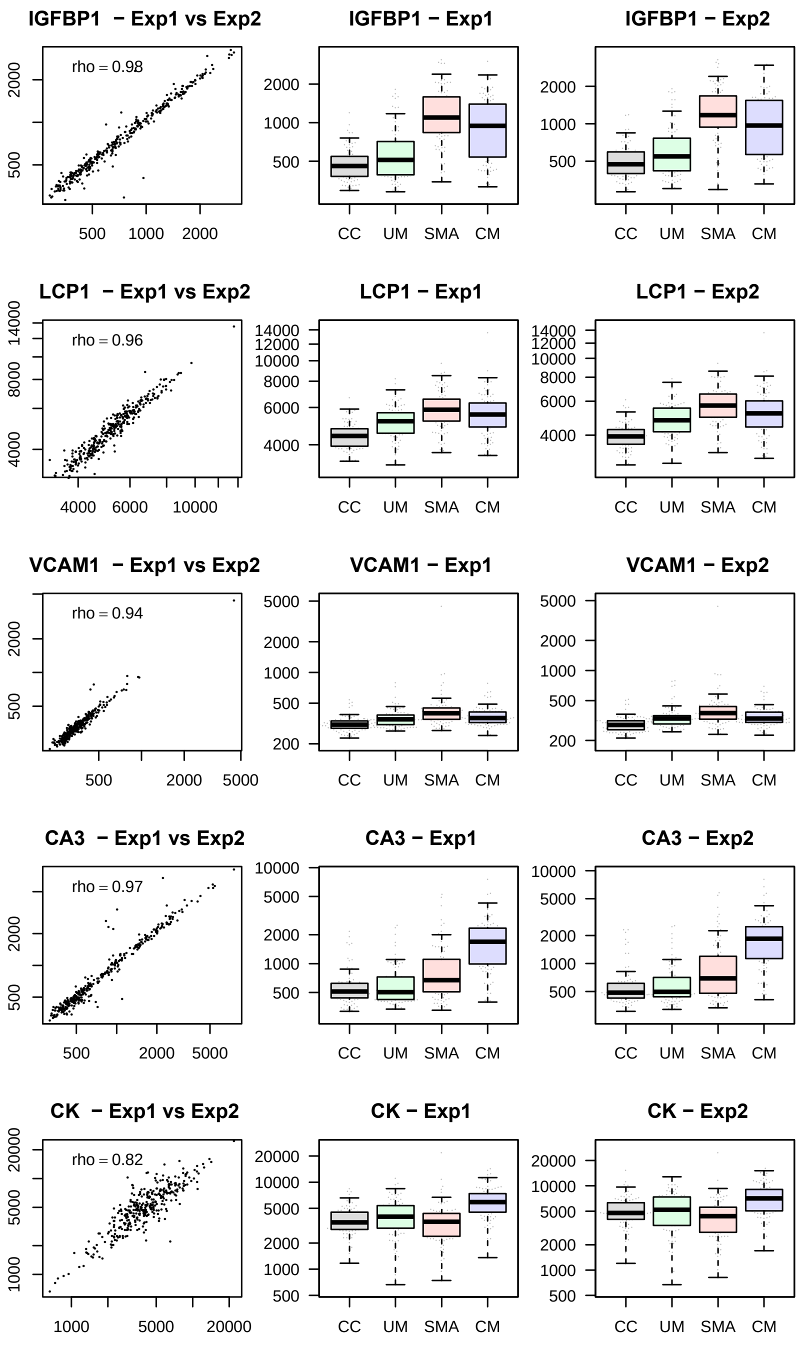
IGFBP1 − Exp1 vs Exp2
rho = 0.98
IGFBP1 − Exp1
IGFBP1 − Exp2
LCP1 − Exp1 vs Exp2
rho = 0.96
LCP1 − Exp1
LCP1 − Exp2
VCAM1 − Exp1 vs Exp2
rho = 0.94
VCAM1 − Exp1
VCAM1 − Exp2
CA3 − Exp1 vs Exp2
rho = 0.97
CA3 − Exp1
CA3 − Exp2
CK − Exp1 vs Exp2
rho = 0.82
CK − Exp1
CK − Exp2
CC UM SMA CM

Figure S3.

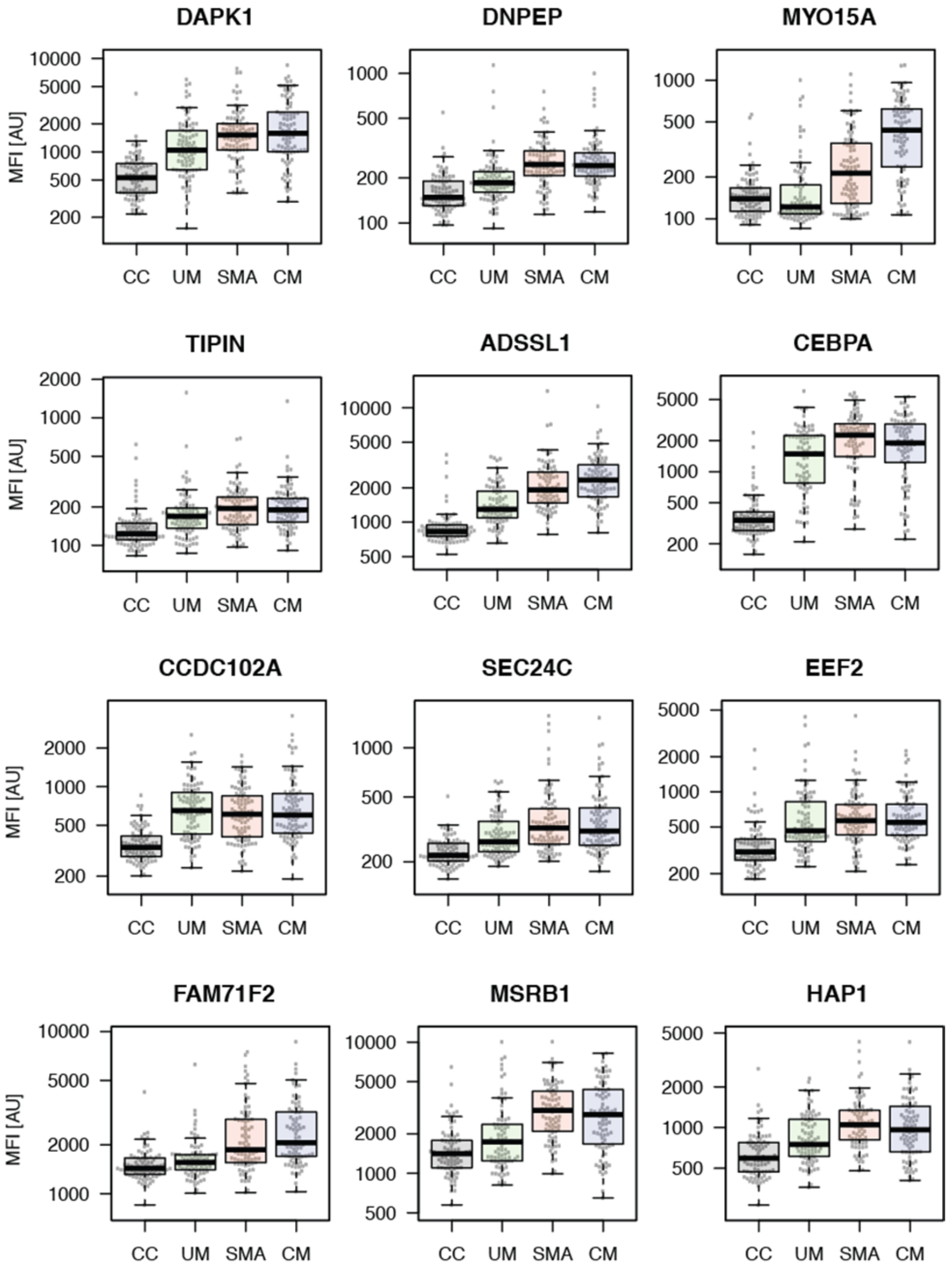
DAPK1
DNPEP
MYO15A
TIPIN
ADSSL1
CEBPA
CCDC102A
SEC24C
EEF2
FAM71F2
MSRB1
HAP1
MFI [AU]
CC
UM
SMA
CM

Figure S4.

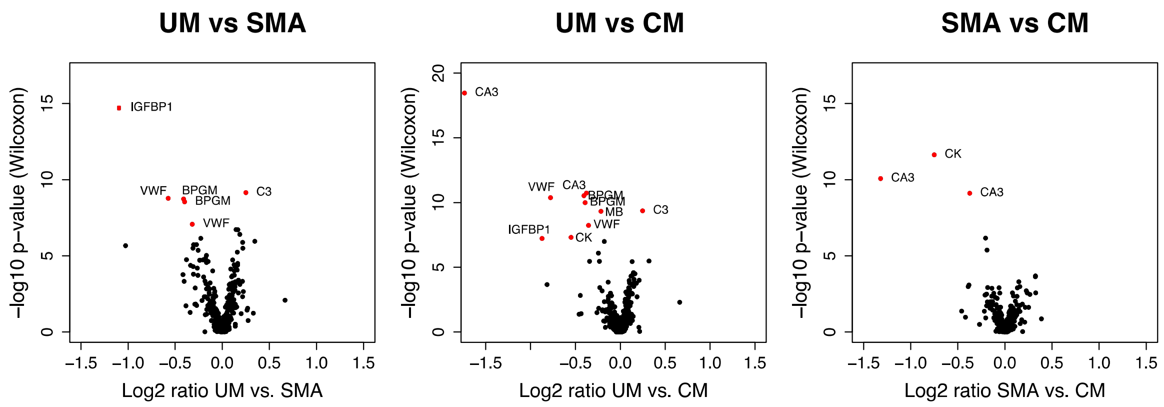
UM vs SMA
UM vs CM
SMA vs CM
−log10 p−value (Wilcoxon)
Log2 ratio UM vs. SMA
Log2 ratio UM vs. CM
Log2 ratio SMA vs. CM
IGFBP1
VWF
BPGM
C3
CA3
MB
CK

Figure S5.

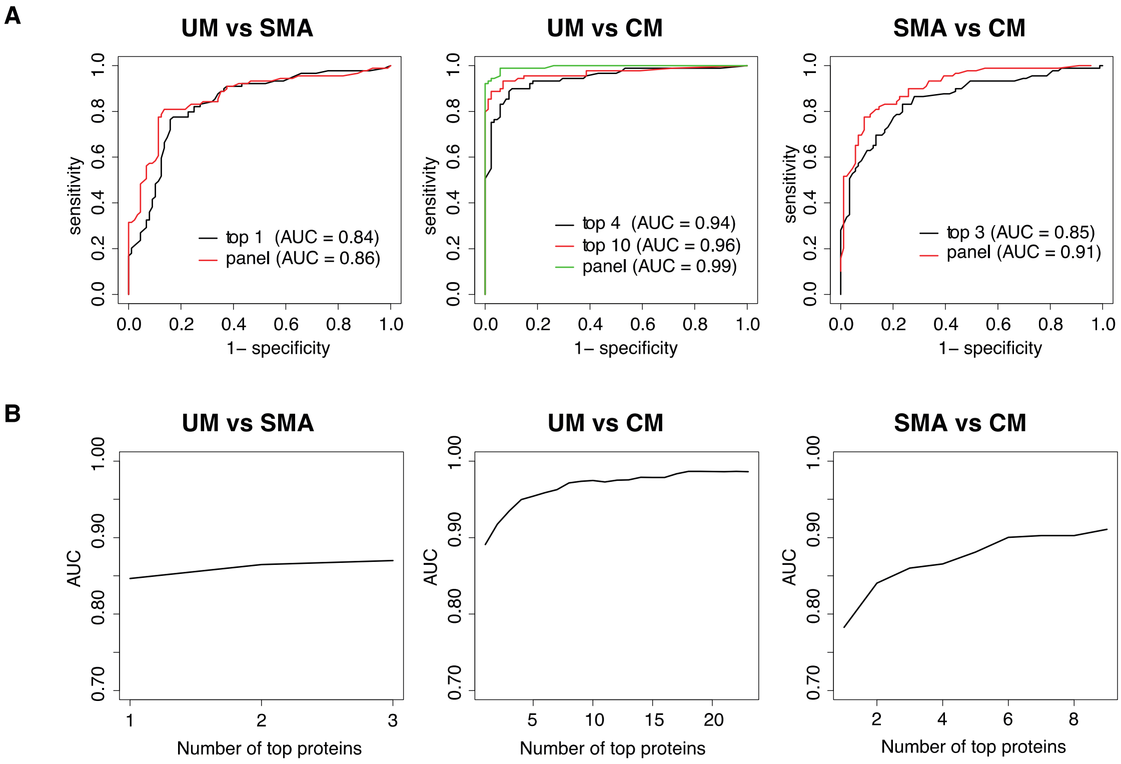
A
UM vs SMA
sensitivity
1– specificity
top 1 (AUC = 0.84)
panel (AUC = 0.86)
UM vs CM
top 4 (AUC = 0.94)
top 10 (AUC = 0.96)
panel (AUC = 0.99)
SMA vs CM
top 3 (AUC = 0.85)
panel (AUC = 0.91)
B
UM vs SMA
UM vs CM
SMA vs CM
AUC
Number of top proteins

Figure S6.

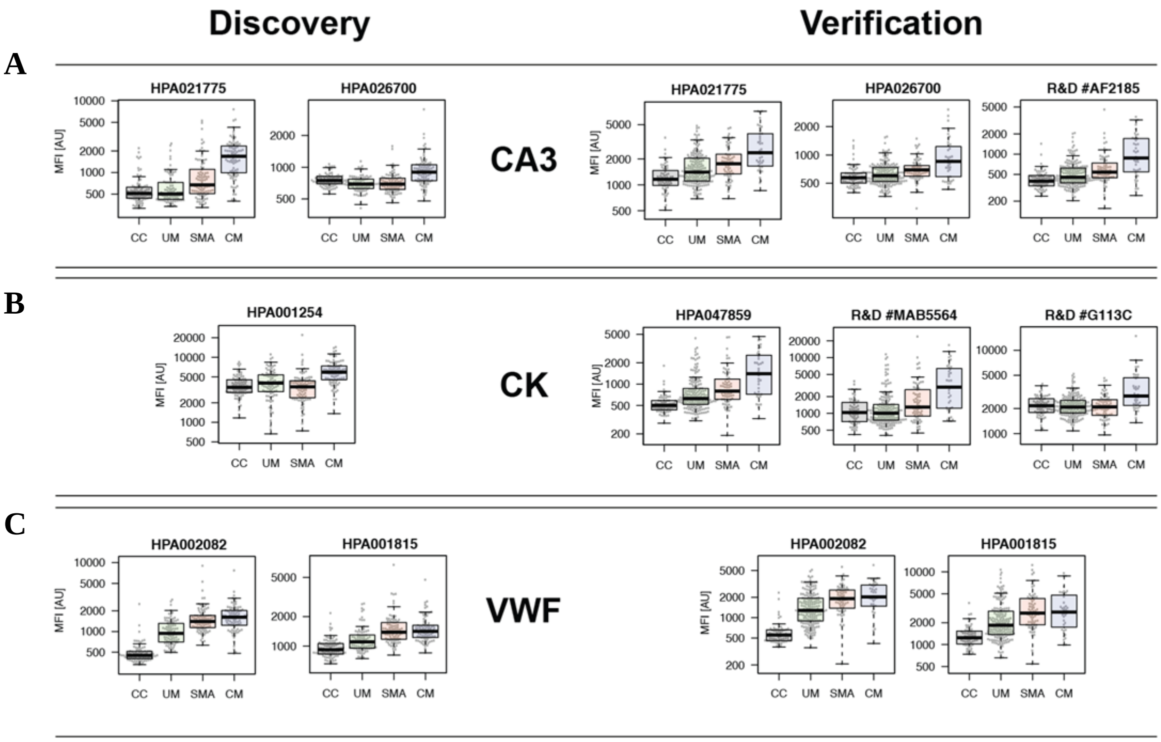

Discovery
Verification
A
HPA021775
HPA026700
CA3
HPA021775
HPA026700
R&D #AF2185
B
HPA001254
CK
HPA047859
R&D #MAB5564
R&D #G113C
C
HPA002082
HPA001815
VWF
HPA002082
HPA001815
MFI [AU]
CC UM SMA CM

Figure S7.

A

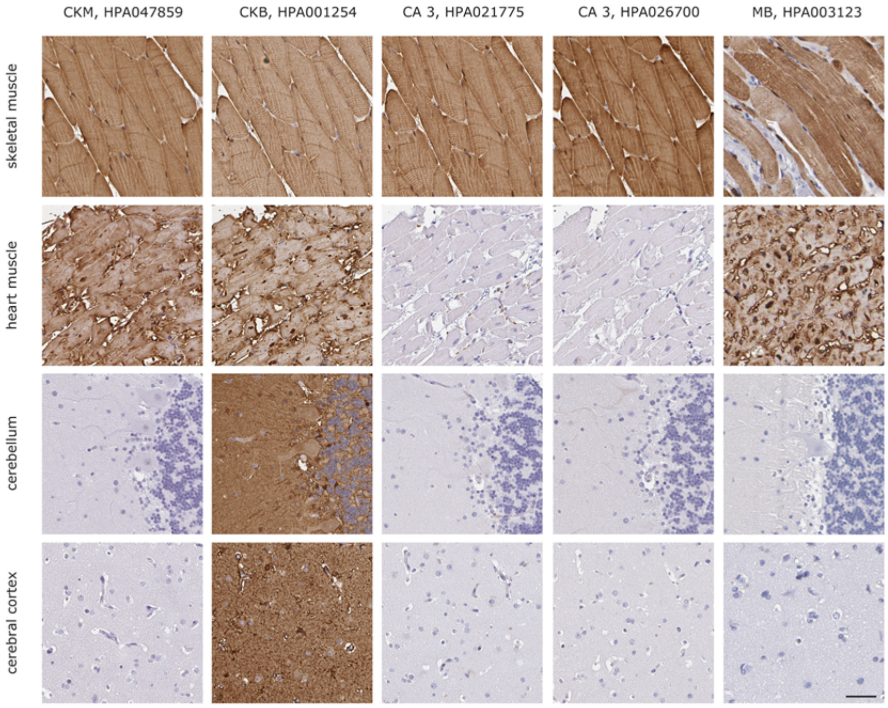

CKM, HPA047859
CKB, HPA001254
CA 3, HPA021775
CA 3, HPA026700
MB, HPA003123
skeletal muscle
heart muscle
cerebellum
cerebral cortex

B

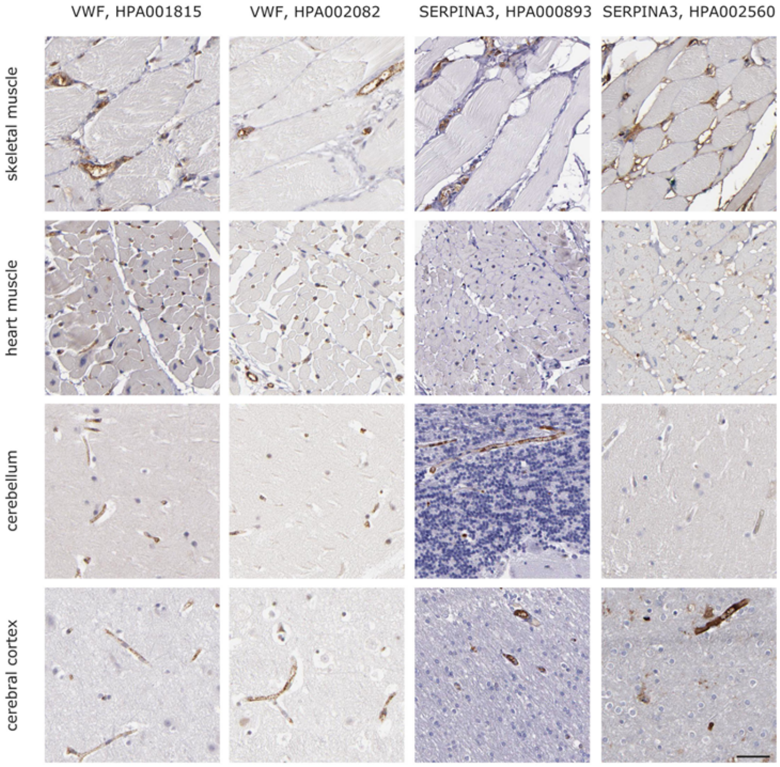

VWF, HPA001815
VWF, HPA002082
SERPINA3, HPA000893
SERPINA3, HPA002560
skeletal muscle
heart muscle
cerebellum
cerebral cortex

Figure S8.

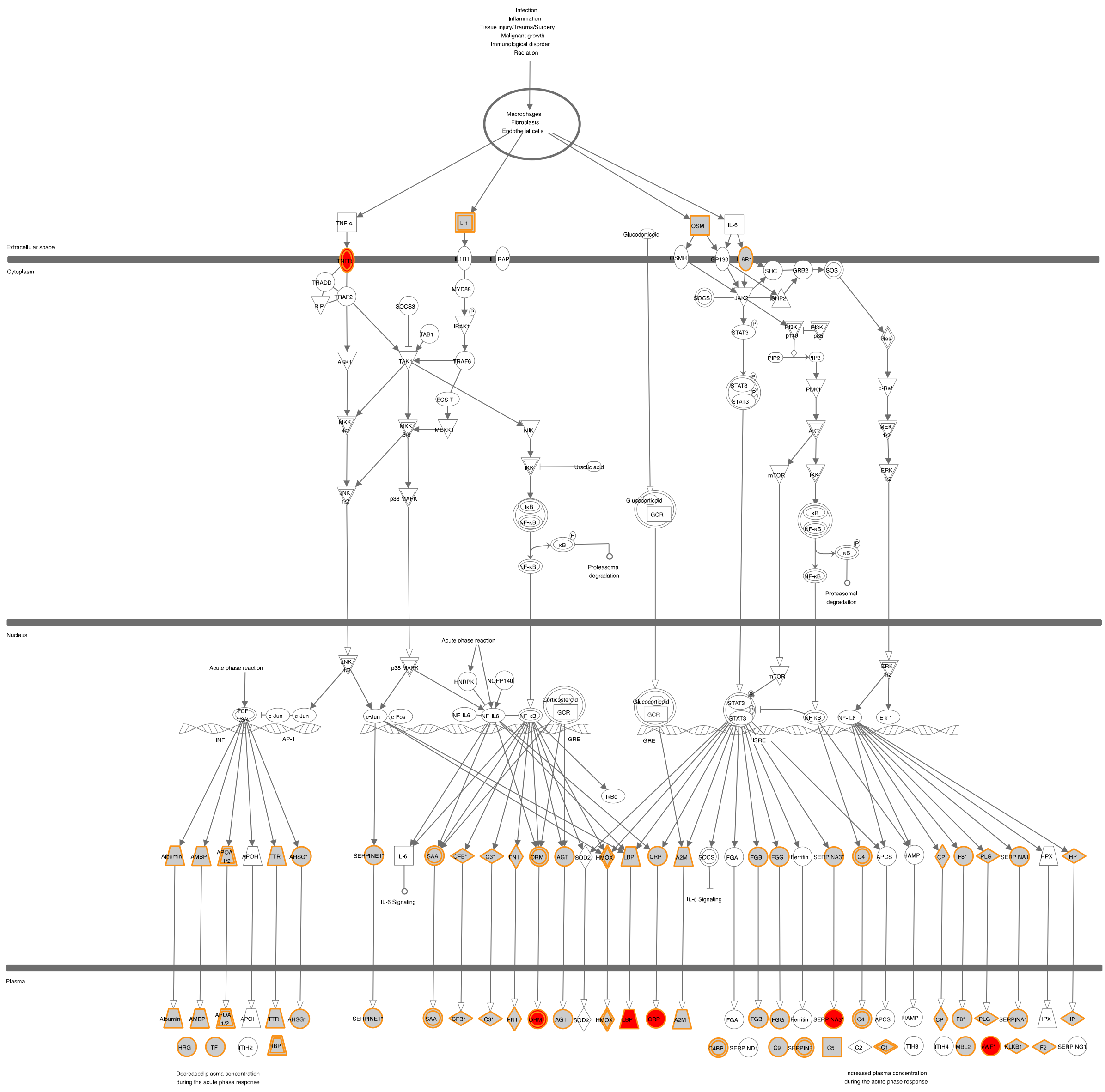
Infection
Inflammation
Tissue injury/Trauma/Surgery
Malignant growth
Immunological disorder
Radiation
Macrophages
Fibroblasts
Endothelial cells
Extracellular space
Cytoplasm
TNF-α
IL-1
OSM
IL-6
Glucocorticoid
TRADD
TRAF2
RIP
SOCS3
TAB1
MYD88
IRAK1
TRAF6
ECSIT
MEKK1
ASK1
TAK1
NIK
IKK
Ursolic acid
IκB
NF-κB
Proteasomal degradation
SHC
GRB2
SOS
SHP2
JAK2
STAT3
PIP2
PIP3
PDK1
AKT
mTOR
Ras
c-Raf
MEK 1/2
ERK 1/2
GCR
Nucleus
Acute phase reaction
HNRPK
NOPP140
Corticosteroid
c-Jun
c-Fos
NF-IL6
Elk-1
HNF
AP-1
GRE
ISRE
IL-6 Signaling
Plasma
Albumin
AMBP
APOA 1/2
APOH
TTR
AHSG*
SERPINE1*
SAA
CFB*
C3*
FN1
ORM
AGT
SOD2
HMOX
LBP
CRP
A2M
SOCS
FGA
FGB
FGG
Ferritin
SERPINA3*
C4
APCS
HAMP
CP
F8*
PLG
SERPINA1
HPX
HP
HRG
TF
ITIH2
RBP
C4BP
SERPIND1
C9
SERPINF
C5
C2
C1
ITIH3
ITIH4
MBL2
KLKB1
F2
SERPING1
Decreased plasma concentration during the acute phase response
Increased plasma concentration during the acute phase response

Figure S9.

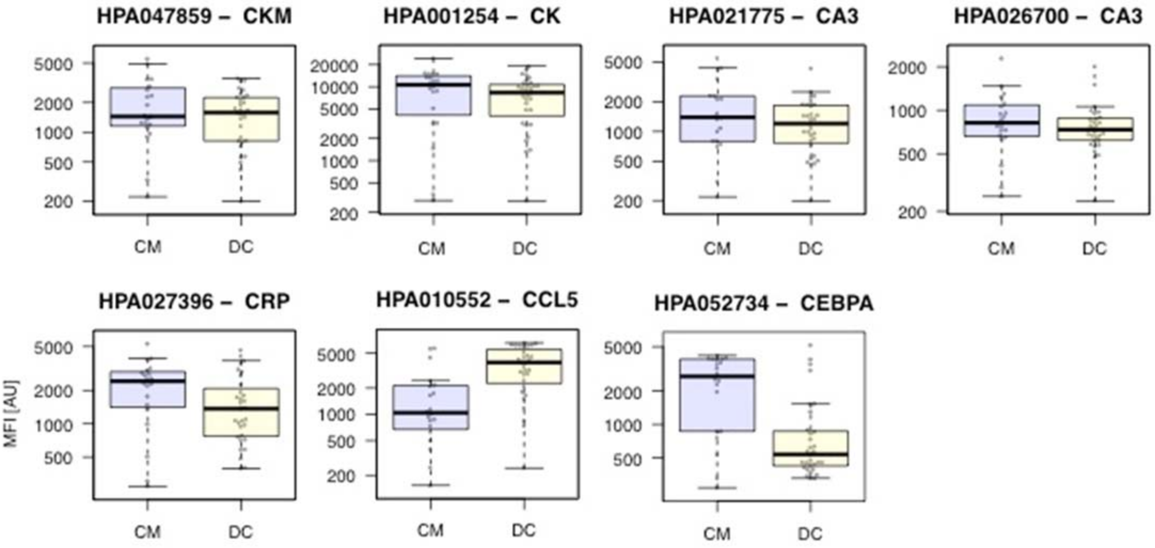
HPA047859 – CKM
HPA001254 – CK
HPA021775 – CA3
HPA026700 – CA3
HPA027396 – CRP
HPA010552 – CCL5
HPA052734 – CEBPA
MFI [AU]
CM
DC

Figure S10.

Supplementary Tables

	Protein	Antibody	p-value (Wilcoxon, Bonf. adj.)
UM vs SMA	IGFBP1	HPA046972	7,10E-13
	C3	HPA020432	2,60E-07
	VWF	HPA002082	6,15E-07
	BPGM	HPA016493	6,80E-07
	VWF	HPA001815	3,10E-05
UM vs CM	CA3	HPA021775	1,28E-16
	CA3*	HPA026700	6,86E-09
	BPGM	HPA016493	1,09E-08
	VWF	HPA002082	1,55E-08
	C3	HPA020432	1,60E-07
	MB	HPA003123	1,76E-07
	VWF	HPA001815	2,15E-06
	CK	HPA001254	1,81E-05
	IGFBP1	HPA046972	2,20E-05
SMA vs CM	CK	HPA001254	8,56E-10
	CA3	HPA021775	3,11E-08
	CA3*	HPA026700	2,87E-07

Table S1.

	Protein	Protein description	Antibody	AUC
**UM vs SMA**	**IGFBP1**	insulin-like growth factor binding protein 1	HPA04697	0,847
	VWF	von Willebrand factor	HPA00208	0,865
	HBA 1,2	hemoglobin, alpha 2, hemoglobin, alpha 1	HPA04378	0,870
**UM vs CM**	**CA3**	carbonic anhydrase III, muscle specific	HPA02177	0,891
	**AGT**	angiotensinogen (serpin peptidase inhibitor, clade A, 8)	HPA00155	0,917
	**FBLN1**	fibulin 1	HPA00161	0,935
	**BPGM**	2,3-bisphosphoglycerate mutase	HPA01649	0,950
	CA3*	carbonic anhydrase III, muscle specific	HPA02670	0,954
	MB	myoglobin	HPA00312	0,959
	IGFBP1	insulin-like growth factor binding protein 1	HPA04697	0,963
	ELANE	elastase, neutrophil expressed	HPA00118	0,972
	FURIN	furin (paired basic amino acid cleaving enzyme)	HPA00590	0,974
	CTSD	cathepsin D	HPA00300	0,975
	CCL17	chemokine (C-C motif) ligand 17	HPA04087	0,973
	GPT	glutamic-pyruvate transaminase	HPA03105	0,975
	CK	creatine kinase	HPA00125	0,975
	HSPG2	heparan sulfate proteoglycan 2	HPA01889	0,979
	ACTN2	actinin, alpha 2	HPA00831	0,979
	IGJ	immunoglobulin J polypeptide	HPA04413	0,979
	TNFSF13B	tumor necrosis factor (ligand) superfamily, member 13b	HPA03054	0,983
	C5	complement component 5	HPA02933	0,987
	F2	coagulation factor II (thrombin)	HPA00735	0,987
	VWF	von Willebrand factor	HPA00208	0,986
	TIE1	tyrosine kinase with immunoglobulin-like and EGF-like	HPA02743	0,986
	ADAMTS13	ADAM metallopeptidase with thrombospondin motif 1, 13	HPA04284	0,986
	PAFAH1B3	platelet-activating factor acetylhydrolase 1b	HPA03563	0,986
**SMA vs CM**	**CA3**	carbonic anhydrase III, muscle specific	HPA02177	0,783
	**CK**	creatine kinase	HPA00125	0,840
	IGFBP1	insulin-like growth factor binding protein 1	HPA04697	0,860
	ITGA2B	integrin, alpha 2b (antigen CD41)	HPA03117	0,866
	CTSD	cathepsin D	HPA00300	0,881
	SAA4*	serum amyloid A4, constitutive	HPA04868	0,900
	NGF	nerve growth factor (beta polypeptide)	HPA05029	0,903
	CALC A,B	calcitonin-related polypeptide beta, alpha	HPA04370	0,903
	FURIN	furin (paired basic amino acid cleaving enzyme)	HPA00590	0,911

* was excluded from verification cohort

Table S2.

**Von Willebrand factor (VWF)**	length	seq	
**HPA002082**	125aa	1379-1503	RITLLLMASQEPQRMSRNFVRYVQGLKKKKVIVIPVGIGPHA NLKQIRLIEKQAPENKAFVLSSVDELEQQRDEIVSYLCDLAPE APPPTLPPDMAQVTV GPGLLGVSTLGPKRNSMVLDVAFVL
**HPA001815**	125aa	1815-1939	ARSNRVTVFPIGIGDRYDAAQLRILAGPAGDSNVVKLQRIEDL PTMVTLGNSFLHKLCSGFVRICMDEDGNEKRPGDVWTLPDQ CHTVTCQPDGQTLLKSHRVNCDRGLRPSCPNSQSPVKVEKT
**Carbonic anhydrase III (CA3)**			
**HPA021775**	76 aa	14- 89	DHWHELFPNAKGENQSPVELHTKDIRHDPSLQPWSVSYDGG SAKTILNNG KTCRVVFDDTYDRSMLRGGPLPGPYR
**HPA026700**	71aa	190-260	YWTYQGSFTTPPCEECIVWLLLKEPMTVSSDQMAKLRSLLSS AENEPPVP LVSNWRPPQPINNRVVRASFK
**Creatine kinase (CK)**			
**HPA001254 (CKB/M)**	145aa	237-381	VISMQKGGNMKEVFTRFCTGLTQIETLFKSKDYEFMWNPHL GYILTCPSNLGTGLRAGVHIKLPNLGKHEKFSEVLKRLRLQKR GTGGVDTAAVGGVFDVSNADRLGFSEVELVQMVVDGVKLLI EMEQRLEQGQAIDDLMPAQK
**HPA047859 (CKM)**	24aa	1-24	MPFGNTHNKFKLNYKPEEEYPDLS

Table S3. Antibody-antigen properties.

Gene	Uniprot	HPA	Univariate hit	Multivariate hit
**A1BG**	P04217	HPA041687		
**A2M**	P01023	HPA002265		
**ABCB7**	O75027	HPA034982		
**ACE2**	Q9BYF1	HPA043894		
**ACTN2**	P35609	HPA008315		(x)
**ADA**	P00813	HPA001399	x	
**ADA**	P00813	HPA023884	x	
**ADAMTS13, ADAMTS13.1**	Q76LX8	HPA042844		(x)
**ADM**	P35318	HPA031806	x	
**AGL**	P35573	HPA028498		
**AGT**	P01019	HPA001557	x	x
**AHSG**	P02765	HPA001524		
**AHSG**	P02765	HPA001525		
**AK5**	Q9Y6K8	HPA019128		
**ALB**	P02768	HPA031025		
**AMBP**	P02760	HPA001497		
**ANG**	P03950	HPA036017		
**ANG**	P03950	HPA036018		
**ANGPT1**	Q15389	HPA018816		
**ANGPT1**	Q15389	HPA018793		
**ANXA13**	P27216	HPA018535		
**ANXA13**	P27216	HPA019569		
**ANXA13**	P27216	HPA019650		
**ANXA5**	P08758	HPA035330		
**APOA1**	P02647	HPA046715		
**APOF**	Q13790	HPA001902		
**APOL1**	O14791	HPA018885		
**APOL5**	Q9BWW9	HPA040968		
**APP**	P05067	HPA001462		
**AREG, AREGB**	P15514	HPA008720		
**ARG2**	P78540	HPA000663		
**ARSB**	P15848	HPA037770		
**ARSB**	P15848	HPA037771		
**ASL**	P04424	HPA016646		
**ATRN**	O75882	HPA008853		
**AXL**	P30530	HPA037422		
**AZGP1**	P25311	HPA012582		
**B2M**	P61769	HPA006361		
**BASP1**	P80723	HPA045218		
**BCHE**	P06276	HPA001560		
**BCL6**	P41182	HPA004899		
**BDNF**	P23560	HPA005774		
**BMP1**	P13497	HPA014572		
**BMP2**	P12643	HPA049966		
**BMP3**	P12645	HPA045344		
**BMP7**	P18075	HPA009264		
**BMP7**	P18075	HPA009158		
**BPGM**	P07738	HPA016493	x	x
**BPGM**	P07738	HPA016493	x	
**BPGM**	P07738	HPA028735		
**C17orf57**	Q8IY85	HPA021633		
**C17orf57**	Q8IY85	HPA026561		
**C1S**	P09871	HPA018852		
**C3**	P01024	HPA003563		
**C3**	P01024	HPA020432		
**C4B, C4A**	P0C0L4	HPA046356		
**C4B, C4A**	P0C0L4	HPA050103		
**C4BPA**	P04003	HPA000926		
**C4BPA**	P04003	HPA001578		
**C5**	P01031	HPA029339		(x)
**C6**	P13671	HPA043823		
**C7**	P10643	HPA001465		
**C8A**	P07357	HPA028217		
**C8A**	P07357	HPA028221		
**C8B**	P07358	HPA023694		
**C9**	P02748	HPA029577		
**CA2**	P00918	HPA001550		
**CA3**	P07451	HPA021775	x	x
**CA3**	P07451	HPA026700	x	x
**CALCB, CALCA**	P10092,	HPA043700		(x)
**CAMP**	P49913	HPA029874		
**CCL1**	P22362	HPA049861		
**CCL11**	P51671	HPA011652		
**CCL14**	Q16627	HPA030268		
**CCL15**	Q16663	HPA046429		
**CCL16**	O15467	HPA042909		
**CCL17**	Q92583	HPA040876		(x)
**CCL18**	P55774	HPA047485		

**CCL19**	Q99731	HPA026849		
**CCL22**	O00626	HPA046252		
**CCL23**	P55773	HPA042015		
**CCL24**	O00175	HPA035631		
**CCL27**	Q9Y4X3	HPA042918		
**CCL28**	Q9NRJ3	HPA046231		
**CCL5**	P13501	HPA010552	x	
**CCL7**	P80098	HPA045228		
**CCL8**	P80075	HPA050280		
**CD14**	P08571	HPA002127	x	
**CD14**	P08571	HPA001887	x	
**CD27**	P26842	HPA038936		
**CD27**	P26842	HPA038937		
**CD33**	P20138	HPA035832		
**CD40**	P25942	HPA031567		
**CD40**	P25942	HPA031568		
**CD40LG**	P29965	HPA045827		
**CD44**	P16070	HPA005785		
**CD55**	P08174	HPA002190		
**CD55**	P08174	HPA024386x		
**CD59**	P13987	HPA026494		
**CD5L**	O43866	HPA026432		
**CD8B**	P10966	HPA029164		
**CD9**	P21926	HPA005677		
**CDH12**	P55289	HPA006309		
**CDH12**	P55289	HPA006310		
**CDH16**	O75309	HPA036260		
**CDH5**	P33151	HPA030562		
**CDH6**	P55285	HPA007456		
**CFB**	P00751	HPA001817		
**CFB**	P00751	HPA001832		
**CFH, CFHR1**	P08603,	HPA038922		
**CFI**	P05156	HPA001143		
**CKB**	P12277	HPA001254	x	x
**CLCF1**	Q9UBD9	HPA042444		
**CNDP1**	Q96KN2	HPA008933		
**CNTF**	P26441	HPA019654		
**COL18A1**	P39060	HPA011025		
**CP**	P00450	HPA001834		
**CPB2**	Q96IY4	HPA004146		
**CPN1**	P15169	HPA040323		
**CPN1**	P15169	HPA040323x		
**CPN2**	P22792	HPA004732		
**CRP**	P02741	HPA027396	x	
**CSF1**	P09603	HPA022244	x	
**CSF2**	P04141	HPA048058		
**CSF3**	P09919	HPA001412		
**CXCL1, CXCL3, CXCL2**	P09341,	HPA043754		
**CXCL10**	P02778	HPA046579		
**CXCL11**	O14625	HPA042298		
**CXCL16**	Q9H2A7	HPA028972		
**CXCL6**	P80162	HPA043269		
**DEFA1, DEFA1B, DEFA3**	P59665,	HPA044693		
**DEFB104B, DEFB104A**	Q8WTQ1	HPA045292		
**EDN1**	P05305	HPA031977		
**EDN1**	P05305	HPA031978		
**EDN3**	P14138	HPA036618		
**EGFR**	P00533	HPA018530x		
**ELANE**	P08246	HPA001184		x
**ENG**	P17813	HPA011862		
**EPB41L2**	O43491	HPA005730		
**EPB41L2**	O43491	HPA006642	x	
**EPO**	P01588	HPA027572		
**EPOR**	P19235	HPA012110		
**EPOR**	P19235	HPA036320		
**F10**	P00742	HPA030629	x	
**F11**	P03951	HPA039808		
**F12**	P00748	HPA003825x		
**F13A1**	P00488	HPA001804		
**F13B**	P05160	HPA003827		
**F2**	P00734	HPA007359		(x)
**F3**	P13726	HPA049292		
**F5**	P12259	HPA002036		
**F7**	P08709	HPA004826		
**F8**	P00451	HPA000594		
**F8**	P00451	HPA000284		
**F8**	P00451	HPA000284		
**F9**	P00740	HPA000254		
**FBLN1**	P23142	HPA001612		
**FBLN1**	P23142	HPA001613	x	x
**FGB**	P02675	HPA001901		
**FGG**	P02679	HPA027529		

**FIGF**	O43915	HPA027342		
**FKBP5**	Q13451	HPA031092		
**FKBP5**	Q13451	HPA031093		
**FLT1**	P17948	HPA011740	x	
**FN1**	P02751	HPA027066		
**FSTL3**	O95633	HPA045378		
**FTMT, FTH1**	Q8N4E7,	HPA043650		
**FURIN**	P09958	HPA005905	x	x
**G6PD**	P11413	HPA000247		
**G6PD**	P11413	HPA000247		
**G6PD**	P11413	HPA000834		
**GAL**	P22466	HPA049864		
**GC**	P02774	HPA001526		
**GDF5**	P43026	HPA015648		
**GP1BA**	P07359	HPA013316		
**GPT**	P24298	HPA031059		(x)
**GPT**	P24298	HPA031060		
**GPX1**	P07203	HPA044758	x	
**HABP2**	Q14520	HPA021074	x	
**HABP2**	Q14520	HPA019518	x	
**HBA2, HBA1**	P69905	HPA043780		(x)
**HBB, HBD, HBG1**	P68871,	HPA043234		
**HFE2**	Q6ZVN8	HPA014472		
**HGF**	P14210	HPA040360		
**HGF**	P14210	HPA044088		
**HIF1A**	Q16665	HPA001275		
**HIF1AN**	Q9NWT6	HPA001179		
**HIF1AN**	Q9NWT6	HPA029260		
**HIF3A**	Q9Y2N7	HPA041141		
**HMGB2, HMGB1**	P09429,	HPA003506		
**HMOX1**	P09601	HPA000635		
**HMOX2**	P30519	HPA040611		
**HP, HPR**	P00738,	HPA047750		
**HRG**	P04196	HPA050269		
**HSPG2**	P98160	HPA018892		(x)
**ICAM1**	P05362	HPA004877		
**IFNA21, IFNA4, IFNA7**	P01568,	HPA047557		
**IFNB1**	P01574	HPA038795		
**IFNB1**	P01574	HPA038794		
**IFNG**	P01579	HPA049525		
**IFNW1, IFNA21, IFNA4**	P05000,	HPA045659		
**IGFBP1**	P08833	HPA046972	x	x
**IGJ**	P01591	HPA044132		(x)
**IL11**	P20809	HPA044955		
**IL12A**	P29459	HPA001886		
**IL12B**	P29460	HPA041100		
**IL16**	Q14005	HPA018467		
**IL17A**	Q16552	HPA045886		
**IL17F**	Q96PD4	HPA028770		
**IL18**	Q14116	HPA003980		
**IL19**	Q9UHD0	HPA049514		
**IL1A**	P01583	HPA030643		
**IL1B**	P01584	HPA044649		
**IL20**	Q9NYY1	HPA031387		
**IL22**	Q9GZX6	HPA023684		
**IL23A**	Q9NPF7	HPA001554		
**IL24**	Q13007	HPA008252		
**IL28B, IL28A**	Q8IZI9,	HPA043017		
**IL29**	Q8IU54	HPA014071		
**IL2RA**	P01589	HPA046738		
**IL3**	P08700	HPA030770		
**IL4**	P05112	HPA042270		
**IL6R**	P08887	HPA001411		
**IL6R**	P08887	HPA047592		
**IL7**	P13232	HPA019590	x	
**IL8**	P10145	HPA045822		
**ITGA2B**	P08514	HPA031170		(x)
**ITGA2B**	P08514	HPA031168		
**ITGA2B**	P08514	HPA031169		
**ITGA3**	P26006	HPA008572		
**ITGA5**	P08648	HPA002642		
**ITGA6**	P23229	HPA027585		
**ITGAE**	P38570	HPA036313		
**ITGAV**	P06756	HPA004856		
**ITGB3**	P05106	HPA027852		
**ITIH1**	P19827	HPA042049		
**ITIH5**	Q86UX2	HPA038304		
**ITIH6**	Q6UXX5	HPA001686		
**KLKB1**	P03952	HPA005634		
**KNG1**	P01042	HPA001645		
**LBP**	P18428	HPA001508	x	
**LCN2**	P80188	HPA002695		

**LCP1**	P13796	HPA019493	x	
**LEP**	P41159	HPA030721		
**LEP**	P41159	HPA030722		
**LIF**	P15018	HPA018844		
**LRG1**	P02750	HPA001888		
**LRG1**	P02750	HPA001889		
**MASP1**	P48740	HPA001617		
**MASP1**	P48740	HPA009641		
**MASP2**	O00187	HPA029314		
**MASP2**	O00187	HPA029313		
**MB**	P02144	HPA003123		x
**MBL2**	P11226	HPA002027		
**MCAM**	P43121	HPA008848		
**MGP**	P08493	HPA013949		
**MIF**	P14174	HPA003868		
**MMP1**	P03956	HPA007713		
**MMP1**	P03956	HPA031456		
**MMP13**	P45452	HPA036107		
**MMP13**	P45452	HPA036106		
**MMP2**	P08253	HPA001939	x	
**MMP3**	P08254	HPA007875x		
**MMP7**	P09237	HPA006991		
**MMP8**	P22894	HPA021221		
**MMP8**	P22894	HPA022935		
**MMP9**	P14780	HPA001238		
**MMP9**	P14780	HPA001238		
**MPL**	P40238	HPA007619		
**MPO**	P05164	HPA021147		
**MSTN**	O14793	HPA018534		
**MSTN**	O14793	HPA021681x		
**NAIP**	Q13075	HPA047000		
**NAIP**	Q13075	HPA042438		
**NAIP**	Q13075	HPA050102		
**NCAM1**	P13591	HPA039835		
**NCAM2**	O15394	HPA030900		
**NEFM**	P07197	HPA023138		
**NEFM**	P07197	HPA022845	x	
**NGF**	P01138	HPA050298		(x)
**NOS1**	P29475	HPA043350		
**NOS2**	P35228	HPA038086		
**NOS2**	P35228	HPA038087		
**NOS3**	P29474	HPA040212		
**NRP1**	O14786	HPA002880		
**NTF3**	P20783	HPA032000		
**ORM1, ORM2**	P02763,	HPA046438		
**ORM1, ORM2**	P02763,	HPA047725	x	
**OSM**	P13725	HPA029814		
**PAFAH1B1**	P43034	HPA020036		
**PAFAH1B3**	Q15102	HPA035639		(x)
**PDGFA**	P04085	HPA045273		
**PDGFB**	P01127	HPA011972		
**PF4V1, PF4**	P10720,	HPA043455		
**PIGR**	P01833	HPA006154		
**PLA2G1B**	P04054	HPA047822		
**PLAT**	P00750	HPA003412		
**PLAU**	P00749	HPA008719		
**PLG**	P00747,	HPA021602		
**POMC**	P01189	HPA046135		
**POSTN**	Q15063	HPA012306		
**PPBP**	P02775	HPA008354		
**PPBP**	P02775	HPA008354		
**PROC**	P04070	HPA005550		
**PROS1**	P07225	HPA007724		
**PROS1**	P07225	HPA023974		
**PROZ**	P22891	HPA016503		
**PTGDS**	P41222	HPA004938		
**RBP4**	P02753	HPA001641		
**REG3G, REG3A**	Q6UW15,	HPA048334		
**RELN**	P78509	HPA046512		
**REN**	P00797	HPA005131		
**CTSD**	P07339	HPA003001		(x)
**S100A1**	P23297	HPA006462		
**S100A4**	P26447	HPA007973		
**S100A7A, S100A7**	Q86SG5,	HPA006997		
**S100A8**	P05109	HPA024372		
**S100A9**	P06702	HPA004193		
**S100B**	P04271	HPA015277		
**SAA4**	P35542	HPA048680		(x)
**SDC1**	P18827	HPA006185		
**SEBOX**	Q9HB31	HPA045689		
**SELP**	P16109	HPA002655		
**SERPINA1**	P01009	HPA001292		

**SERPINA3**	P01011	HPA000893	x	
**SERPINA3**	P01011	HPA002560	x	
**SERPINA6**	P08185	HPA017864		
**SERPINC1**	P01008	HPA001816		
**SERPINC1**	P01008	HPA024007		
**SERPINE1**	P05121	HPA001539		
**SERPINE1**	P05121	HPA024527		
**SERPINE2**	P07093	HPA000277		
**SERPINE2**	P07093	HPA000277		
**SERPINF1**	P36955	HPA005825		
**SERPINF2**	P08697	HPA001885		
**SLC12A3**	P55017	HPA028748	x	
**SLC40A1**	Q9NP59	HPA011754		
**SOD1**	P00441	HPA001401		
**SPP1**	P10451	HPA005562		
**SPP1**	P10451	HPA027540		
**TF**	P02787	HPA001527		
**TFPI**	P10646	HPA005575		
**TFR2**	Q9UP52	HPA011937		
**TFRC**	P02786	HPA028598		
**TGFA**	P01135	HPA042297		
**TGFB2**	P61812	HPA006373		
**TGFB2**	P61812	HPA049818		
**TGFB3**	P10600	HPA001129		
**TGFBI**	Q15582	HPA008612		
**THPO**	P40225	HPA042965		
**THPO**	P40225	HPA048828		
**TIE1**	P35590	HPA007243		
**TIE1**	P35590	HPA027432		(x)
**TIMP2**	P16035	HPA001764		
**TIMP2**	P16035	HPA049145		
**TIMP3**	P35625	HPA001139		
**TLR9**	Q9NR96	HPA004731		
**TNFAIP1**	Q13829	HPA013333		
**TNFAIP1**	Q13829	HPA014641		
**TNFRSF1A**	P19438	HPA019119		
**TNFRSF1B**	P20333	HPA004796	x	
**TNFRSF8**	P28908	HPA014720		
**TNFSF11**	O14788	HPA008128		
**TNFSF13B**	Q9Y275	HPA030546		(x)
**TTR**	P02766	HPA005150		
**UTS2**	O95399	HPA017000		
**VCAM1**	P19320	HPA001618	x	
**VCAM1**	P19320	HPA034796	x	
**VEGFB**	P49765	HPA044361		
**VWF**	P04275	HPA001815	x	
**VWF**	P04275	HPA002082	x	x
**XCL2, XCL1**	Q9UBD3,	HPA047690		

Table S4.

Methods S1

Clinical data and sample collection. A 2.5 ml blood sample was obtained from each participant in an EDTA blood collection tube for subsequent plasma separation. Blood samples were kept on ice and transferred to the central malaria laboratory. Plasma for this study was harvested by centrifugation (1000 *g*, 10 minutes), aliquoted and frozen at -80°C no later than 4 hours following collection. The total of freeze/thaw cycles was limited and did not exceed 3 times during the entire study.

Biotinylation of plasma samples. Biotinylation was performed as previously described [1] (refer to Supplementary Methods). Briefly, plasma samples were diluted using a liquid handler (SELMA, CyBio). Primary amino groups of plasma proteins were labeled using N-hydroxysuccinimide ester-biotin with a polyethylene oxide spacer (NHS-PEO4-Biotin, Pierce) as biotinylation reagent following the manufacturer's instructions. After incubation for 2 h, the reaction was stopped by the addition of 1 M Tris–HCl, pH 8.0 and samples were stored at −20°C. Two samples of the discovery cohort and four samples of the verification cohort had to be removed due to technical failure.

Quality assessment of suspension bead-array. To analyze the technical variation of the array, the initial screening using the 'targeted array' including 356 samples and 384 antibodies was reproduced in an independent experiment. The median coefficient of variation (CV) of sample replicates (n=12) within one experiment (intra-assay) was 5.5% for normalized data (Fig. S1A). To evaluate the inter-assay variation, the relative intensities between the two independent experiments were compared (Fig. S1B). The sample-wise median correlation coefficient was 0.992 ranging from 0.94 to 0.99. To assess the reproducibility also antibody-wise, the relative intensities of each antibody were compared in the two experiments. The correlation coefficients of antibodies ranged from 0.45 to 0.99 with a median of 0.89. Patient- and antibody-wise correlation coefficients were additionally determined for the 24 duplicated patient samples in discovery and validation cohort with the top 30 proteins. The median correlation coefficient for antibody-wise correlations was 0.67 and for sample-wise correlations 0.64 due to the low number of samples (Fig. S1C).

In general, many substantial differences ($p < 0.001$) in protein profiles were found between the patient groups when utilizing the 'targeted array'. In order to confirm that the observed

differences were specific to the pre-selected set of proteins in the ‘targeted array’, profiling with ‘random arrays’ was conducted. To obtain an overview of the observed differences in the ‘targeted array’ and the ‘random arrays’ principal component analysis (PCA) was performed, which provides a visualization tool for information-rich data by reducing the dimensionality. By projecting the data into the first few principal components, separations between disease groups can be observed dependent on the variables (proteins). Interestingly, the PCA of the ‘targeted array’ indicated a more spread separation by the proteins contained in this array compared to the ‘random array’ (Fig. S2A). Hence, the observed large differences were specific to the selected antibodies.

Immunohistochemistry. Immunohistochemical analysis was performed on healthy adult Human tissues as described before according to the Human Protein Atlas (HPA) standard operating procedures [2]. All incubations were performed at room temperature and reagents were from Thermo Fisher Scientific, unless specified. Briefly, prior to immunostaining, deparaffinization and hydration in xylene and graded ethanol to distilled water using a Leica Autostainer XL (Leica Biosystems, Wetzlar, Germany) were performed. Endogeneous peroxidase was blocked using 0.3 % H_2O_2 in 95% ethanol for 5 minutes. Pressure boiling (Decloaking chamber, Biocare Medical, Walnut Creek, CA) with retrieval buffer pH6 for 4 minutes at 125°C was carried out to perform heat induced epitope retrieval (HIER). After HIER, slides were allowed to cool to 90°C. Immunohistochemistry was performed using an Autostainer 480 (Thermo Fisher Scientific, Waltham, MA, USA). Between procedures described below, sections were washed in wash buffer twice. To block cross reactivity, incubation with UV block for 5 minutes was performed. Rabbit polyclonal antibodies affinity-purified using the antigen as affinity-ligand [3] and secondary labeled horse radish peroxidase-polymer were incubated for 30 minutes, respectively. Thereafter 3,3´-Diaminobenzidine (DAB) was used for detection for 10 minutes. Counterstaining was performed using hematoxylin (Histolab, Järfälla, Sweden) for 5 minutes and then the slides were rinsed in tap water and lithium carbonate water, diluted 1:5 from saturated solution, in total 10 min. Finally, the slides were dehydrated in graded ethanol. Stained slides were scanned using an automated high-resolution slide scanner (Aperio Technologies, Vista, CA, USA).

Self-organizing tree algorithm (SOTA) analysis. The SOTA algorithm combines the advantages of both hierarchical clustering and Self-Organizing Maps (SOM). Briefly, it picks

a node with the largest diversity and splits it into two nodes[4]. The tree was allowed to grow for 4 cycles. To visualize the high dimensional array data set we applied principal components analysis (PCA) using the R function prcomp[5-7]. Thereby, the dimensionality of the data set was reduced while maintaining as much variance as possible. Calculation is done by a singular value decomposition of the scaled and centered data matrix.

Multivariate analysis. Several logistic regression models with different variables (sex, age proteins) were fitted. In order to achieve a robust result, in each model hundred fits with different random seeds were performed. Based on the resulting λ_1-value in each fit, the average λ_1 was used to fit the model. Because the variables age and sex did not provide large contribution to the classification, the analysis was continued using only proteins as variable for classification. The resulting protein panels as well as the corresponding area under the ROC curve (AUC) of each model is presented.

L1-penalized logistic regression model

Multiclass classification and at the same time performing feature (e.g., proteins) selections commonly faced in many biological applications is not a trivial problem. One way to deal with multiclass (i.e., UM, SMA and CM) classification is to decompose the classification into several binary classification tasks (one-vs.-one). Then, on each binary classification, binary classifiers are obtained using a binary classification method, i.e., L_1 penalized-logistic regression.

Logistic regression is a supervised method for binary classification [8]. Because it is a simple, flexible and straightforward model that is easy to extend, the extensions of logistic regression have been widely used in genomics and proteomics research [9, 10].

In high-dimensional datasets such as an array, where usually variables are in similar or higher numbers than observations are available, and where the variables are correlated (multicollinearity), the classical logistic regression would perform badly and provide inaccurate estimates. It could result in a perfect fit to the data with no bias and high variance, which would lead to inaccurate predictions. In order to prevent this problem, a penalty for complexity in the model was introduced.

In our case, yi is an array of two malaria disease subtypes (i.e., cerebral malaria (CM), severe malarial anemia (SMA), or uncomplicated malaria (UM)), and xj is the intensity level of the jth protein. We can fit the following logistic model:

$$\log it(p(x_i)) = \beta_0 + \sum_{j=1}^{m} \beta_j x_j$$

where p(x) is the probability of success (i.e., y = 1) given x. To fit this model, the following log-likelihood had to be optimized:

$$\ell(\beta) = \sum_{j=1}^{n} \{ y_i \log(p(x_i)) + (1 - y_i) \log(1 - p(x_i)) \}$$

To penalize logistic regression, we can impose a penalty as defined in the following penalized log-likelihood:

$$\ell^*(\beta) = \ell(\beta) - \lambda J(\theta)$$

where $J(\theta)$ is a penalty function. There are several penalty functions being proposed, and in this study we implement the ℓ1 penalization proposed by Tibshirani [11] and known as Lasso. The ℓ1 penalization use

$$J(\theta) = \sum_{i=1}^{n} \|\beta_i\|$$

as the penalty function. The Lasso shrinks all regression coefficients β toward zero and set some of them to zero. Hence, it performs parameter estimation and variable selection at the same time. For a defined value of λ, the challenge is to optimize the penalized log-likelihood. Goeman [12] proposed the *full gradient* algorithm for maximizing Lasso-penalized log-likelihood. The choice of the tuning parameter λ is crucial. Since the *full gradient* algorithm finds the estimates of regression coefficients for a fixed value of λ, the optimal value of λ is found by a cross-validation procedure. In this procedure, cross-validated partial log-likelihood for a range of λ was calculated and the λ value, which gives the maximum log-likelihood is selected using Brent's algorithm [13]. These algorithm and procedures are implemented in an R package called penalized [12].

Note that the sample partitioning in the cross-validation procedure are performed randomly, which can lead to slightly different λ values for different random seed numbers. To obtain the more reliable λ, 100 cross-validations are performed with different random seed numbers and the obtained (λ1, λ2, ..., λ100) are averaged ($\bar{\lambda}$). The final ℓ1-penalized model is then fitted based on the $\bar{\lambda}$.

References

1. Neiman M, Hedberg JJ, Donnes PR, et al. Plasma profiling reveals human fibulin-1 as candidate marker for renal impairment. *J Proteome Res* 2011; **10**(11): 4925-34.
2. Kampf C, Olsson I, Ryberg U, Sjostedt E, Ponten F. Production of tissue microarrays, immunohistochemistry staining and digitalization within the human protein atlas. *Journal of visualized experiments : JoVE* 2012; (63).
3. Nilsson P, Paavilainen L, Larsson K, et al. Towards a human proteome atlas: high-throughput generation of mono-specific antibodies for tissue profiling. *Proteomics* 2005; **5**(17): 4327-37.
4. Herrero J, Valencia A, Dopazo J. A hierarchical unsupervised growing neural network for clustering gene expression patterns. *Bioinformatics* 2001; **17**(2): 126-36.
5. Venables WNaBDR. Modern Applied Statistics with S: Springer-Verlag; 2002.
6. Mardia KV, J. T. Kent, and J. M. Bibby. Multivariate Analysis: London: Academic Press; 1979.
7. Becker RA, Chambers, J. M. and Wilks, A. R. The New S Language: Wadsworth & Brooks/Cole; 1988.
8. Hosmer DW, Lemeshow, S. Applied Logistic Regression. New York, NY: Wiley; 1989.
9. Liao JG, Chin KV. Logistic regression for disease classification using microarray data: model selection in a large p and small n case. *Bioinformatics* 2007; **23**(15): 1945-51.
10. Sun H, Wang S. Penalized logistic regression for high-dimensional DNA methylation data with case-control studies. *Bioinformatics* 2012; **28**(10): 1368-75.
11. Tibshirani R. Regression shrinkage and selection via the lasso: a retrospective. *J R Stat Soc B* 2011; **73**: 273-82.
12. Goeman JJ. L1 penalized estimation in the Cox proportional hazards model. *Biometrical journal Biometrische Zeitschrift* 2010; **52**(1): 70-84.
13. Brent RP. Algorithms for Minimization without Derivatives: Englewood Cliffs: Prentice-Hall; 1973.
